# The limits of precision monomer placement in chain growth polymerization

**DOI:** 10.1038/ncomms10514

**Published:** 2016-02-01

**Authors:** Guillaume Gody, Per B. Zetterlund, Sébastien Perrier, Simon Harrisson

**Affiliations:** 1 Department of Chemistry, University of Warwick, Warwick CV4 7AL, UK; 2 Centre for Advanced Macromolecular Design, School of Chemical Engineering, University of New South Wales, Sydney, New South Wales 2052, Australia; 3 Faculty of Pharmacy and Pharmaceutical Sciences, Monash University, Parkville, Victoria 3052, Australia; 4 Laboratoire des Interactions Moléculaires et Réactivité Chimique et Photochimique, CNRS UMR5623, Université Paul Sabatier Toulouse III, 118 Route de Narbonne, Toulouse 31062, France

## Abstract

Precise control over the location of monomers in a polymer chain has been described as the ‘Holy Grail' of polymer synthesis. Controlled chain growth polymerization techniques have brought this goal closer, allowing the preparation of multiblock copolymers with ordered sequences of functional monomers. Such structures have promising applications ranging from medicine to materials engineering. Here we show, however, that the statistical nature of chain growth polymerization places strong limits on the control that can be obtained. We demonstrate that monomer locations are distributed according to surprisingly simple laws related to the Poisson or beta distributions. The degree of control is quantified in terms of the yield of the desired structure and the standard deviation of the appropriate distribution, allowing comparison between different synthetic techniques. This analysis establishes experimental requirements for the design of polymeric chains with controlled sequence of functionalities, which balance precise control of structure with simplicity of synthesis.

## Abstract (web summary)

Chemists increasingly seek to control monomer sequencing in aperiodic copolymers. Here, the authors show that the statistical nature of chain growth strongly limits the achievable control, and establish parameters for polymer design that balance precise control with simplicity of synthesis.

## Graphical abstract

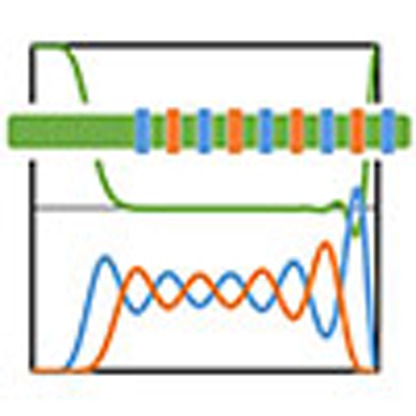

How precisely can we control the location of a monomer in a synthetic polymer? This question is crucial to the synthesis of aperiodic copolymers: ‘copolymers in which monomer sequence distribution is not regular but follows the same arrangement in all chains'^1^. Included in this definition are polymers that contain an ordered sequence of distinct blocks, and polymers that contain specific monomers at precisely defined locations^2^,^3^,^4^. Such polymers could serve as a medium for information storage, as does DNA, or catalyse complex reactions, as do enzymes^5^. But for these applications to be realized, precise control over structure is essential, and any deviation from the desired structure must be quantified.

If only small quantities of polymer are required, near perfect control over structure can be obtained using stepwise synthesis on a solid^6^,^7^ or soluble polymer^8^,^9^ support, as in peptide synthesis. For the rapid preparation of moderate to large quantities of well-defined polymer, however, we must turn to living or controlled chain growth polymerizations^10^,^11^,^12^,^13^,^14^,^15^,^16^,^17^,^18^,^19^,^20^,^21^,^22^,^23^. But unlike stepwise techniques, chain growth polymerization is a statistical process that produces a distribution of products. In this paper, we establish a lower bound to the structural variation that exists even under ideal conditions of living polymerization. Our results are applicable to all chain polymerizations, which are carried out under living or near-living conditions, including ring-opening metathesis polymerization (ROMP)^24^, the various types of reversible deactivation radical polymerization (RDRP, also known as controlled/‘living' radical polymerization)^25^, and ionic polymerizations^26^,^27^.

The production of aperiodic copolymers requires control over the arrangement of monomers, which should be replicated in all chains. The degree of control required can be interpreted with varying degrees of severity, corresponding to the sequence-defined, multisite and multiblock copolymers considered by Lutz^1^ (Fig. 1): (a) control over the absolute position of a monomer with respect to the chain end, or the absolute separation between two monomers (sequence-defined copolymers); (b) control over the relative position of a monomer with respect to the total chain length, or the relative separation between two monomers (multisite copolymers) or (c) control over the number and order of blocks in a multiblock copolymer, without regard to the distribution of each individual block (multiblock copolymers).

The level of structural control that is actually achieved can be quantified in two ways: in terms of the fraction of chains that correspond to the desired structure (the yield), and in terms of the standard deviation, *σ*, of the distribution. High yields and low standard deviations imply high levels of control. In this article, we propose the standard deviation of the monomer distribution as a quantitative measure of precision; derive simple expressions for these distributions under ideal chain growth polymerization conditions and apply these expressions to a number of recent examples of multisite and multiblock copolymers. Our results demonstrate that the level of control over monomer placement is quite limited, even under ideal conditions, and that multiblock copolymers comprised of many short blocks will inevitably contain a large proportion of defective chains.

## Results

### Absolute position of monomers

Absolute control over the location of an individual monomer is the most demanding goal of polymer synthesis. However, techniques based on chain-growth polymerization lead, even in the best-controlled polymerizations, to a distribution of structures. For very short chains, separation of a single oligomeric structure may be possible, but requires extensive use of chromatography^28^,^29^.

Under ideal conditions of living polymerization, the degree of polymerization obeys a Poisson distribution^30^, whose probability mass function is given in Table 1 (full derivations of all results in Table 1 may be found in Supplementary Note 1). In reality, unavoidable side reactions such as termination and chain transfer result in deviations from the Poisson distribution. The ideal is most closely approached by anionic polymerization^31^, but this technique must be carried out in dry, nonprotic solvents and is incompatible with most functional groups. RDRP techniques^32^,^33^,^34^,^35^,^36^,^37^,^38^,^39^ tolerate a wide range of functionalities, but lead to broader distributions because of termination reactions and the addition of multiple monomers per activation/deactivation cycle.

The highest level of control over monomer placement achievable using chain growth polymerization techniques is obtained by addition of a non-homopolymerizing monomer (*B*), such as maleic anhydride, to a polymer of average length *n* ({*A*_*n*_}), followed by subsequent chain extension^10^,^11^,^12^,^13^. As *B* cannot homopolymerize, only one monomer of *B* will be added to each chain. Use of an excess of *B* ensures that one monomer of *B* will be added to every chain, giving the average structure {*A*_n_}*B*_1_. In a variation on this technique, addition of B may occur by a step-growth reaction, such as via azide-alkyne coupling, followed by chain extension from a suitable functional group^40^,^41^.

Numbering the monomers from the start of the chain, the expected position of *B* is *n*+1. The probability that *B* will be found in this position is approximately 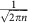 and the standard deviation of the distribution is 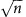. For example, in a polymer with average structure {*A*_10_}*B*_1_{*C*_10_}, only 12.5% of the chains will contain monomer *B* as the 11th unit, while in 26.6% of the chains, *B* will appear before position 8 or after position 14.

In aperiodic copolymers prepared by sequential polymerizations of monomers of similar reactivity^14^,^15^,^16^,^17^,^18^,^19^,^20^, or by addition of alternating^21^,^22^ or very rapidly polymerizing^23^ monomers at intervals during the polymerization, all blocks are Poisson-distributed, at best. Thus, an {*A*_10_*B*_1_*C*_10_} polymer will include chains with no *B* units, as well as chains that contain 2 or more^5^,^42^. The proportion of chains that contain a unit of *B* in position 11 (the midpoint) can be calculated by excluding all chains that contain 11 or more units of *A* (41.7% of chains), as well as all chains in which the sum of units of *A* and *B* is <11 (46.0% of chains). The 12.3% of chains that remain are not significantly fewer than the corresponding proportion of {*A*_10_}*B*_1_{*C*_10_}. In {*A*_10_}*B*_1_{*C*_10_}, however, each polymer chain contained a single copy of *B*. In {*A*_10_*B*_1_*C*_10_}, only 4.6% of chains contain one and only one unit of *B* in position 11.

**Figure 2:**
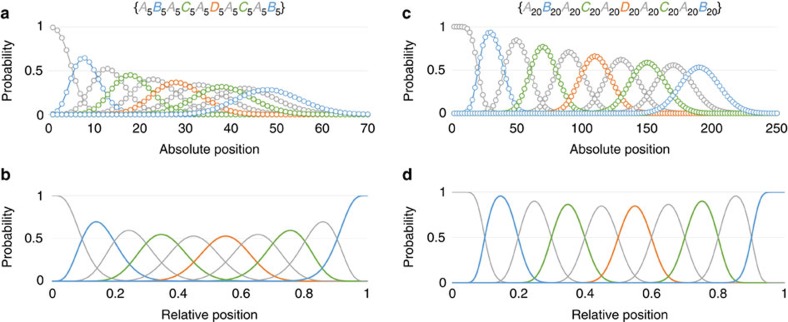
Monomer distributions. Probability of finding monomers from each block of an {*A*_5_*B*_5_*A*_5_*C*_5_*A*_5_*D*_5_*A*_5_*C*_5_*A*_5_*B*_5_} (**a**,**b**) or {*A*_20_*B*_20_*A*_20_*C*_20_*A*_20_*D*_20_*A*_20_*C*_20_*A*_20_*B*_20_} (**c**,**d**) decablock copolymer as a function of absolute position (**a**,**c**) and relative position (**b**,**d**) along the polymer chain.

In general, the probability that the *k*th monomer of a multiblock copolymer is part of the *n*th block is given by the difference between two Poisson cumulative distribution functions (Table 1). As the average position of a block moves further along the polymer chain, its location becomes more diffuse. Shorter blocks are affected to a greater extent than longer blocks, as shown by Fig. 2a,c, which show the location distributions of each block of two *ABACADACAB* decablock copolymers. Those of the short blocks broaden rapidly and overlap significantly. The distributions of the longer blocks broaden more slowly and overlap less, but fewer changes in composition are possible in a given length of polymer. The standard deviation of the location of the block (Table 1) provides a quantitative measure of the attainable precision: in {*A*_10_*B*_1_*C*_10_}, the expected location of *B* is 11.5, with a standard deviation of 3.3; *B* occurs before position 8 or after position 14 in 29.2% of chains.

### Relative position of monomers

Although absolute control over monomer placement is the ideal, it would often be acceptable to control only the relative position of a monomer with respect to the total chain length. In a diblock copolymer, {*A*_n_*B*_m_}, comprising two Poisson-distributed blocks, the relative length of the *A* block with respect to the entire polymer chain is closely approximated by a Beta(*n*,*m*) distribution (see Supplementary Figs 1 and 9 and Supplementary Note 2). The beta distribution is one of the most widely used distributions in statistics, and is often used to model random variables that are limited to a finite range, such as world cloud cover^43^ or the distribution of genetic variation^44^.

In the {*A*_10_}*B*_1_{*C*_10_} copolymer, the distribution of *B* can be approximated as the distribution of the interface between *A* and *C* blocks in an {*A*_10_*C*_10_} diblock copolymer: a Beta(10,10) distribution with expected value 0.50 and standard deviation 0.11. Thus, in ∼30% of the chains, we expect to find *B* outside the relative length range of 0.39–0.61—that is, outside the central fifth of the polymer. In general, the distribution of the relative location of a single inserted monomer narrows as the total length of the polymer increases, and is broadest when the monomer is inserted at the midpoint of the chain (Supplementary Fig. 2).

In an {*A*_n_*B*_m_*C*_p_} copolymer whose inserted block {*B*_m_} is also Poisson distributed, the probability that a monomer of type *B* will be found in a given relative position is given by the difference between two cumulative beta distribution functions with parameters (*n*, *m*+*p*) and (*n*+*m*, *p*), respectively (Table 1). Thus, in the {*A*_10_*B*_1_*C*_10_} copolymer, the expected relative position of the *B* block is 0.50, and its standard deviation is 0.11—almost identical to that of the {*A*_10_}*B*_1_{*C*_10_} copolymer. Only 17.6% of chains contain a monomer of type *B* at the midpoint. Doubling the average length of the *B* block ({*A*_10_*B*_2_*C*_10_}) increases the proportion of chains that contain a monomer of type *B* at the midpoint to 33.6%, but the length must be increased to 11 ({*A*_10_*B*_11_*C*_10_}) to ensure that 95% of the chains contain a monomer of type *B* at the midpoint. The effects of average block length and position on relative position distributions are illustrated in Fig. 2 for two decablock copolymers.

The effect of Poisson broadening on the control that can be achieved over monomer location can be demonstrated using two recently reported multisite polymers^21^. The polymers were prepared by successive additions of the non-homopolymerizing monomers *N*-benzyl maleimide (Bz-MI), *N*-propyl maleimide (Pr-MI) and *N*-pentafluorophenyl maleimide (PFP-MI) to a single electron transfer living radical polymerization (SET-LRP) of styrene with targeted 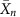 of 65 (ref. 21). For the first polymer, maleimide solutions were added at intervals during the polymerization corresponding to 28, 46 and 70% styrene conversion, leading to the insertion of short segments of copolymer containing on average 1 unit of Bz-MI, Pr-MI or PFP-MI. The second polymer was prepared using a lower initial styrene to initiator ratio and delaying the addition of each type of maleimide until the styrene had reached high conversion. After each maleimide had been fully incorporated, additional styrene was added to generate the intervening polystyrene segments. The effect was to halve the average length of the segments containing Bz-MI and Pr-MI, from 6 in the first synthesis to 3 in the second synthesis. The average length of the PFP-MI block was 3 in both syntheses.

The distribution of each maleimide in the polymer was then calculated, making use of the following simplifying assumptions: (i) all segments were assumed to follow a Poisson distribution; (ii) the maleimides were assumed to be evenly distributed within their respective segments. In this way, the approximate distribution of each maleimide could be obtained by calculating the distribution of each maleimide-containing segment, then normalizing with respect to the segment's maleimide content. Differences in homo- and cross-propagation rate constants of styrene and maleimide were not explicitly considered, as it was assumed that in all cases, the rate of reversible deactivation was much greater than that of propagation. If this assumption is invalid (for example, as a result of very rapid cross-propagation occurring at a comparable rate to deactivation) then broader distributions are obtained.

**Figure 3:**
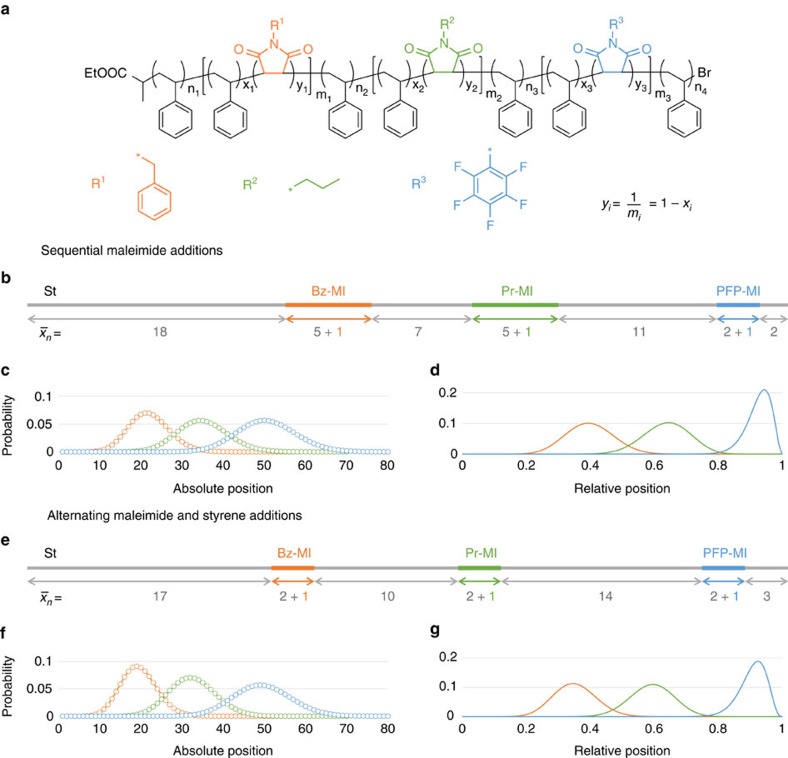
Comparison of multisite copolymers. (**a**) containing benzyl maleimide (Bz-MI), propyl maleimide (Pr-MI) and pentafluorophenyl maleimide (PFP-MI) insertions, prepared using maleimide additions only (**b**–**d**) or by alternating additions of maleimide and styrene (**e**–**g**) to an SET-LRP polymerization of styrene^21^. The expected structures of each copolymer (**a**,**b**,**e**) consist of short regions containing a single maleimide unit, dispersed along a polystyrene chain. The distribution of each type of maleimide is shown in terms of absolute position (**c**,**f**) and position relative to the total chain length (**d**,**g**).

Despite the much shorter segments obtained in the second synthesis, the maleimide distributions are nearly identical (Fig. 3 and Supplementary Table 2). Their standard deviations are determined primarily by the length of the surrounding blocks, with only a small contribution from the length of the block itself. The calculated distributions underestimate the true degree of structural variation, as a Poisson-distributed copolymer with 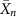 of 53 has a dispersity of 1.02, whereas the measured dispersities of the copolymers were 1.16 (sequential maleimide additions) and 1.27 (alternating maleimide and styrene additions).

We have applied similar analyses to an icosablock copolymer prepared by sequential reversible addition-fragmentation chain transfer (RAFT) polymerizations^14^ (Supplementary Fig. 3), a multisite copolymer prepared by sequential additions of *exo*-functionalized norbornene monomers to the ROMP polymerization of an *endo*-functional norbornene monomer (Supplementary Fig. 4)^23^, and two undecablock copolymers prepared by sequential anionic polymerizations (Supplementary Fig. 5)^15^,^16^. In the RAFT and ROMP copolymers, the use of multiple short blocks (typical length 3 and 2, respectively) results in poorly defined structures. The much longer blocks obtained using anionic copolymerization (minimum block lengths 37 (ref. 15) and 130 (ref. 16)) allow greater control over the relative position of a block within the chain.

### Existence of blocks in multiblock copolymers

The least restrictive goal of aperiodic polymer synthesis is simply to prepare a multiblock copolymer in which each polymer chain contains the desired number of blocks, in the correct order. If, for example, a block of monomer A encoded 1, a block of monomer B encoded 0 and monomer C served to separate consecutive blocks of A or B, a binary string could be represented using a multiblock copolymer, even without specifying the length of each block. The only condition to be fulfilled is that every chain must contain at least one monomer unit from each block.

The probability that a Poisson-distributed block of average length *l* contains at least one monomer is 1–*e*^−*l*^. For a polymer composed of *n* blocks, the proportion of chains that contain all blocks is obtained by multiplying the probabilities for each block. If all blocks are nominally of equal length, the minimum block length, *l*_min_, required for a given proportion, *α*, of chains to contain all blocks is proportional to ln(*n*).


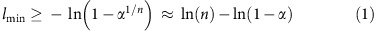


Thus, polymers consisting of many short blocks will contain a significant proportion of defective chains (see Supplementary Figs 3 and 4 for details). Attempts to insert a block of average length 3 will fail in ∼5% of chains; in a copolymer containing 18 such blocks, the majority of chains will be defective (Supplementary Fig. 3). Doubling the targeted block length to 6 results in an exponential decrease in the proportion of missing blocks, which would allow a 20-block copolymer to be prepared with only 5% of defective chains (see Supplementary Fig. 6 for details). These theoretical results were confirmed in a real system by quantifying the residual level of unreacted RAFT agent, corresponding to chains of length zero, after preparation of a series of oligomers of 4-acryloyl morpholine of average degree of polymerization 3–6 (see Supplementary Fig. 7 for details).

**Figure 4:**
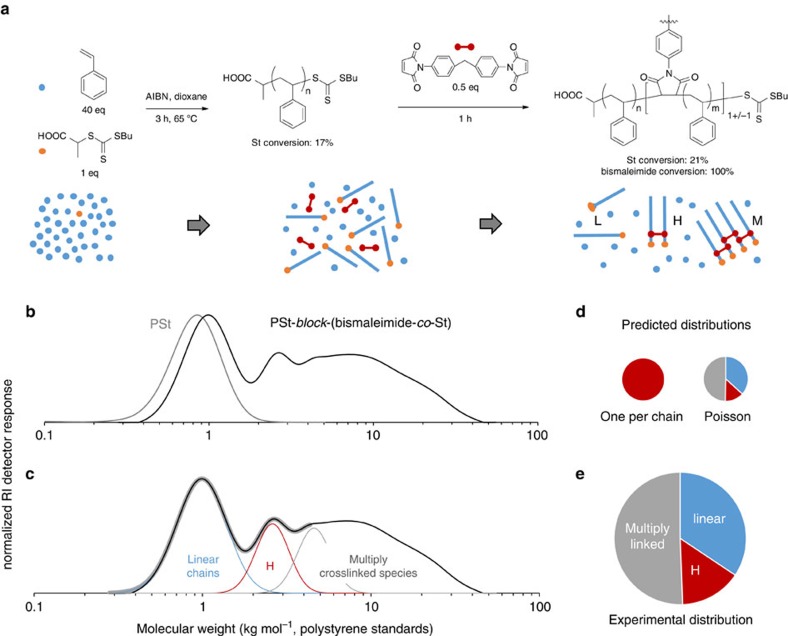
Addition of bismaleimide to a RAFT polymerization of styrene (St). (**a**) After addition of the bismaleimide (0.5 equiv), polymerization was allowed to continue for 1 h, during which time the conversion of St increased from 17 to 21% and the bismaleimide was completely consumed. The resulting copolymer contained, on average, 1 maleimide unit per polystyrene chain (with an estimated standard deviation of 1). The distribution of maleimide units resulted in the formation of a mixture of linear chains (L), double chains (H) and multiply linked structures (M). (**b**) Distribution of chain lengths obtained after addition of 0.5 equiv. of bismaleimide. Linear chains and multiply linked structures are present in addition to H. The distribution immediately before bismaleimide addition is also shown. Deconvolution (**c**) of the low molar mass portion of the peak using Gaussian components (thick grey line represents best fit) allows estimation of the relative mass fractions of each structure (**e**), which are in good agreement with the predicted distribution assuming that the maleimide content of each chain follows a Poisson distribution (**d**).

The validity of the Poisson distribution as a model for the number of units incorporated per chain was further demonstrated by adding a bismaleimide, bis(4-maleimidophenyl)methane, to the early stages of a RAFT polymerization of styrene (Fig. 4). The ratio of bismaleimide to RAFT agent was 1:2, such that on average one maleimide unit was added per chain. The polymerization was allowed to continue for 1 h, during which time the maleimide was entirely consumed. If exactly one maleimide had been added per chain, only the structure H shown in Fig. 4 would be obtained, with twice the molar mass of its constituent polystyrene chains. In fact, size exclusion chromatography (SEC) of the resulting polymer revealed single polymer chains (L), the linked structure (H) and aggregates of multiple chains (M). Deconvolution of the low molar mass portion of the chromatogram allowed the proportions of L and H species to be estimated at 34.4% and 15.1%, respectively. The expected proportions for a Poisson-distributed number of maleimides per chain are 36.8% L and 13.5% H. We have assumed that no looped structures are formed—a more detailed analysis taking this possibility into account can be found in Supplementary Note 3, but leads to a similar level of non-functionalized chains.

This result conflicts with previous reports that only 10% of chains contain no maleimide when an average of 1 maleimide is incorporated per chain^42^,^45^. That value was obtained from a matrix-assisted laser desorption/ionization–time of flight mass spectrum in which polystyrene species containing 1 or 2 units of Bz-MI formed the most intense peaks^45^, while species containing zero or more than 3 units of Bz-MI accounted for 10% and 15% of the sequence distribution, respectively^42^. This implies that the average number of maleimides per chain is greater than 1, however, and so it seems likely that the frequency of zero-maleimide chains was underestimated. The incorporation of a polar maleimide unit may affect the ability of the polymer to form a charged complex, which can be observed by mass spectroscopy, biasing the results.

These results, applied to the SET-LRP multisite polymers of Fig. 3 (ref. 21), suggest that the yield of copolymer chains that contain at least one of each type of maleimide is only 25%, whereas 5% of chains contain no maleimide at all. Better results are obtained for the ROMP multisite polymer^23^ (Supplementary Fig. 4): the use of, on average, two functional monomers per site yields 56% of chains with all four types of functional monomer (the yield of chains with all nine blocks, including spacer blocks, is only 20%). A further increase in average block length to 3, as has been recommended by Lutz and co-workers^42^,^46^, raises the yield of chains containing all four functionalities to over 81%, whereas an average block length of 6 allows the formation of 20-block copolymers with 95% fidelity (Supplementary Fig. 6).

## Discussion

The results presented in this communication assume the ideal case of Poisson-distributed polymer segments, an approximation that has been validated for anionic polymerization, but which does not hold for all controlled chain-growth polymerization techniques. Side reactions such as radical coupling and chain transfer will lead to broader distributions, as will the addition of more than one monomer during each activation–deactivation cycle in RDRP. In such cases, more realistic distributions could be obtained through the use of simulations, or using alternate distribution functions such as the negative binomial distribution which allow for greater variability. An example is given in Supplementary Note 4 for a multiblock polymer with overall dispersity of 1.1 (see also Supplementary Figs 10–14, Supplementary Table 3). The idealized results presented here are valuable because they show the maximum level of precision that can be obtained using chain polymerization, and provide indications of how that precision can be maximized.

We have quantified the maximum level of control over monomer placement that can be obtained using controlled chain growth polymerization techniques. The most ambitious goal of aperiodic polymer synthesis is precise control over the absolute position or separation of introduced monomers. We have shown that, regardless of the technique used, chain growth polymerization is poorly suited to this task, as uncertainty in the position of a given monomer grows in proportion to the square root of the length of the polymer chain. The less ambitious goal of controlling relative position with respect to the total chain length is more achievable, with improved control achieved for longer chains, and as the desired functionality approaches either chain end. Finally, if one desires simply that all blocks of a multiblock copolymer be present in a large majority of chains, this is possible so long as the individual blocks are of sufficient length. In all cases, we have given simple formulae for the probability distributions, expected values and standard deviations of the positions of inserted blocks.

Our research clearly shows the limitations of chain polymerization in the synthesis of very precisely controlled structures—the synthetic analogues of DNA or enzymes. Fortunately, the natural world provides numerous examples of less precisely controlled structures—proteins such as mucins, collagens and elastins—which play vital physiological roles. These proteins are characterized by complex yet variable structures that present multiple functionalities. In many cases, structural complexity enables self-assembly, generating fibrils or gels via a bottom-up process. It is these biomolecules that should provide the inspiration for precision chain polymerization.

Indeed, self-assembly of complex multiblock polymer architectures prepared via chain polymerization has already been used to develop biomimetic stress-stiffening gels^47^, and thermoresponsive, flower-like micelles which display multiple functionalities^14^. A recent review by Bates *et al.*^48^ highlights the enormous range of phase-separated structures that can be generated by polymers containing a relatively small number of blocks; faced with a bewildering array of possibilities, the challenge is to select the architecture which will give the desired properties. Having done so, it is equally important to know the precision with which that architecture can be prepared. This knowledge will allow the design of a new family of materials that offer an optimal balance of precision in the placement of functionalities and simplicity of production, for a wide range of chemical, biomedical and engineering applications.

## Methods

### Chemicals and reagents

Tetrahydrofuran (THF, Ajax Finechem, 99%), *N*,*N*-dimethylformamide (DMF, Merck, HPLC-grade), dimethyl 2,2′-azobis(2-methylpropanoate) (V-601), 2,2′-Azobis[2-(2-imidazolin-2-yl)propane]dihydrochloride (VA-044, Wako), 1,4-dioxane (Sigma-Aldrich, ≥99%) and 4-acryloylmorpholine (NAM, Sigma-Aldrich, 97%) were used without further purification. Styrene (Sigma-Aldrich, ≥99%) was filtered through a basic aluminium oxide (activated, basic, Brockmann I, standard grade, ∼150 mesh, 58A) column before use. All polymerizations were carried out under a nitrogen atmosphere. The RAFT agent, 2-(((butylthio)carbonothiolyl)thio)propanoic acid was prepared according to a reported procedure^49^.

### NMR spectroscopy

^1^H NMR spectra were recorded on Bruker DPX-300 spectrometer using deuterated solvents obtained from Aldrich. Chemical shift values (*δ*) are reported in p.p.m. The residual proton signal of the solvent was used as internal standard.

### Size exclusion chromatography

Number-average molar masses (*M*_n,SEC_) and dispersity values (*Ð*) were determined using THF-SEC performed on an Agilent 390-MDS, comprising of an autosampler and a PLgel 5.0 μm bead-size guard column (50 × 7.5 mm^2^), followed by two linear 5.0 μm bead-size PLgel Mixed D columns (300 × 7.5 mm^2^) and a differential refractive index detector using THF (2% (v/v), TEA) as the eluent at 30 °C with a flow rate of 1 ml min^−1^. The SEC system was calibrated with linear PS EasiVial standards (Agilent Ltd.) ranging from 162 to 105 g mol^−1^. All samples were passed through 0.45 μm PTFE filter before SEC analysis. Molar masses were obtained by conventional calibration using ASTRA software.

### RAFT polymerization of styrene with a controlled addition of bismaleimide

2-(((Butylthio)carbonothiolyl)thio)propanoic acid (114 mg, 0.480 mmol, 1 equiv.), styrene (2 g, 19.2 mmol, 40 equiv.), V-601 (11 mg, 0.048 mmol, 0.1 equiv.) and DMF (0.540 ml) were placed in a flask equipped with a magnetic stirring bar. The tube was then sealed with a rubber septum, degassed with nitrogen and then immersed in an oil bath thermostated at 65 °C. A degassed solution of bismaleimide (91 mg, 0.240 mmol, 0.5 equiv.) in 0.630 ml of DMF was added with a degassed syringe 3 h after the polymerization started (17% styrene conversion at this stage). After 1 more hour of polymerization, a sample was taken for NMR and THF-SEC analysis to ensure the full incorporation of the bismaleimide in the growing copolymer chain (around 4% of styrene conversion took place in this interval). The THF-SEC chromatogram shows multiple bismaleimide insertion.

### RAFT homopolymerization of NAM

*For a targeted degree of polymerization (*X_n_*) of 3*. RAFT agent (281 mg, 1.181 mmol, 1 equiv.), NAM (0.5 g, 3.541 mmol, 3 equiv.), VA-044 (3.82 mg, 0.012 mmol, 0.01 equiv.), dioxane (0.477 ml) and H_2_O (0.257 ml) were placed in a flask equipped with a magnetic stirring bar. The tube was then sealed with a rubber septum, degassed with nitrogen and then immersed in an oil bath thermostated at 70 °C. After 2 h, a sample is withdrawn from the polymerization medium for ^1^H NMR (in dimethylsulphoxide (DMSO-*d*_6_)). Conversion was determined by ^1^H NMR to be 94%, corresponding to an 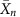 of 2.82.

*For a targeted *X*_n_ of 4*. RAFT agent (211 mg, 0.885 mmol, 1 equiv.), NAM (0.5 g, 3.541 mmol, 4 equiv.), VA-044 (2.20 mg, 0.007 mmol, 0.0077, equiv.), dioxane (0.441 ml) and H_2_O (0.294 ml) were placed in a flask equipped with a magnetic stirring bar. The tube was then sealed with a rubber septum, degassed with nitrogen and then immersed in an oil bath thermostated at 70 °C. After 2 h, a sample was withdrawn from the polymerization medium for ^1^H NMR (in DMSO-*d*_6_). Conversion was determined by ^1^H NMR to be 97%, corresponding to an 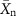 of 3.89.

*For a targeted *X*_n_ of 5*. RAFT agent (169 mg, 0.708 mmol, 1 equiv.), NAM (0.5 g, 3.541 mmol, 5 equiv.), VA-044 (1.53 mg, 0.005 mmol, 0.007 equiv.), dioxane (0.404 ml) and H_2_O (0.331 ml) were placed in a flask equipped with a magnetic stirring bar. The tube was then sealed with a rubber septum, degassed with nitrogen and then immersed in an oil bath thermostated at 70 °C. After 2 h, a sample is withdrawn from the polymerization medium for ^1^H NMR (in DMSO-*d*_6_). Conversion was determined by ^1^H NMR to be 98%, corresponding to an 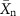 of 4.92.

*For a targeted *X*_n_ of 6*. RAFT agent (141 mg, 0.590 mmol, 1 equiv.), NAM (0.5 g, 3.541 mmol, 6 equiv.), VA-044 (0.95 mg, 0.003 mmol, 0.005 equiv.), dioxane (0.367 ml) and H_2_O (0.367 ml) were placed in a flask equipped with a magnetic stirring bar. The tube was then sealed with a rubber septum, degassed with nitrogen and then immersed in an oil bath thermostated at 70 °C. After 2 h, a sample is withdrawn from the polymerization medium for ^1^H NMR (in DMSO-*d*_6_). Conversion was determined by ^1^H NMR to be 99%, corresponding to an 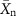 of 5.92.

## Additional information

**How to cite this article:** Gody, G. *et al.* The limits of precision monomer placement in chain growth polymerization. *Nat. Commun.* 7:10514 doi: 10.1038/ncomms10514 (2016).

## Supplementary Material

Supplementary Information Supplementary Figures 1-14, Supplementary Tables 1-3, Supplementary Notes 1-4 and Supplementary References

## Figures and Tables

**Figure 1:**
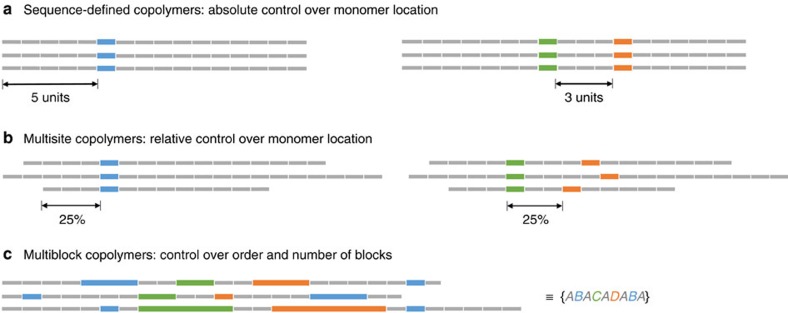
Some aims of precision polymer synthesis. (**a**) Absolute control of monomer position and/or separation in a sequence-defined copolymer. (**b**) Relative control of monomer position and/or separation in a multisite copolymer. (**c**) Control of number and order of blocks in a multiblock copolymer. Throughout this paper, the letters *A*, *B*, *C* and *D* refer to different types of monomer. A collection of polymer chains containing on average *n* units of *A* followed by *m* units of *B* is represented as {*A*_n_*B*_m_}, whereas a single polymer chain containing exactly *N* units of *A* followed by *M* units of *B* is represented as *A*_N_*B*_M_.

**Table 1:**
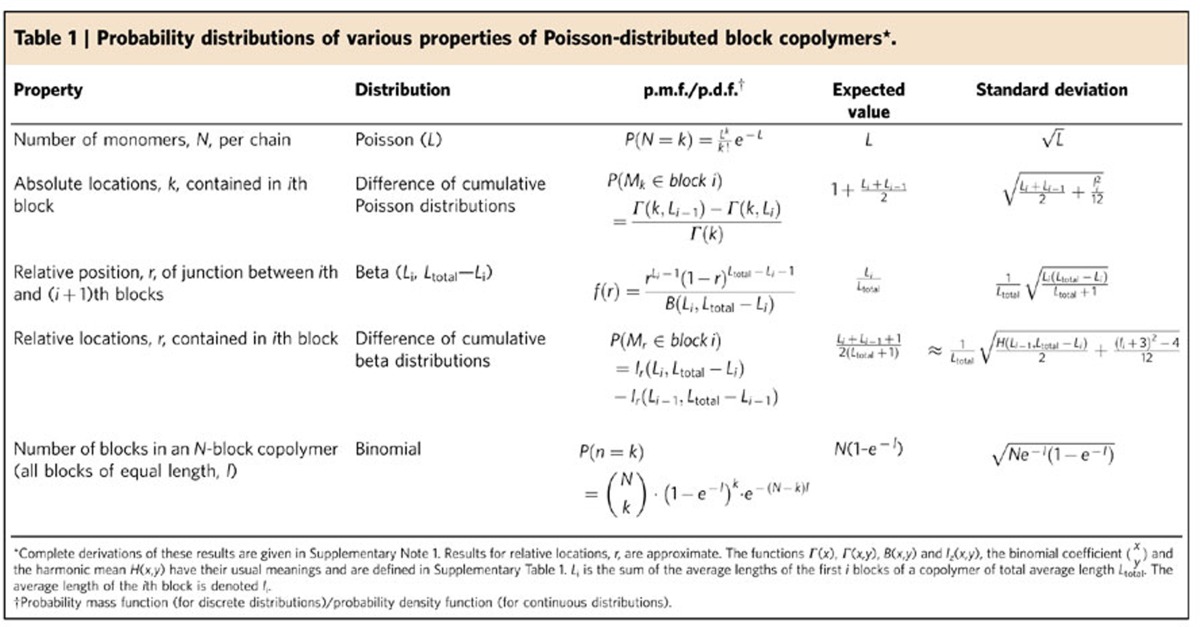
Probability distributions of various properties of Poisson-distributed block copolymers^*^.
