# Supplementary material for: The limits of precision monomer placement in chain growth polymerization
Source: Nat Commun. 2016 Feb 1;7:10514. doi: 10.1038/ncomms10514 (PMC4740409; doi:10.1038/ncomms10514)
Supplement: Supplementary Information — Supplementary Figures 1-14, Supplementary Tables 1-3, Supplementary Notes 1-4 and Supplementary References [file ncomms10514-s1.pdf]

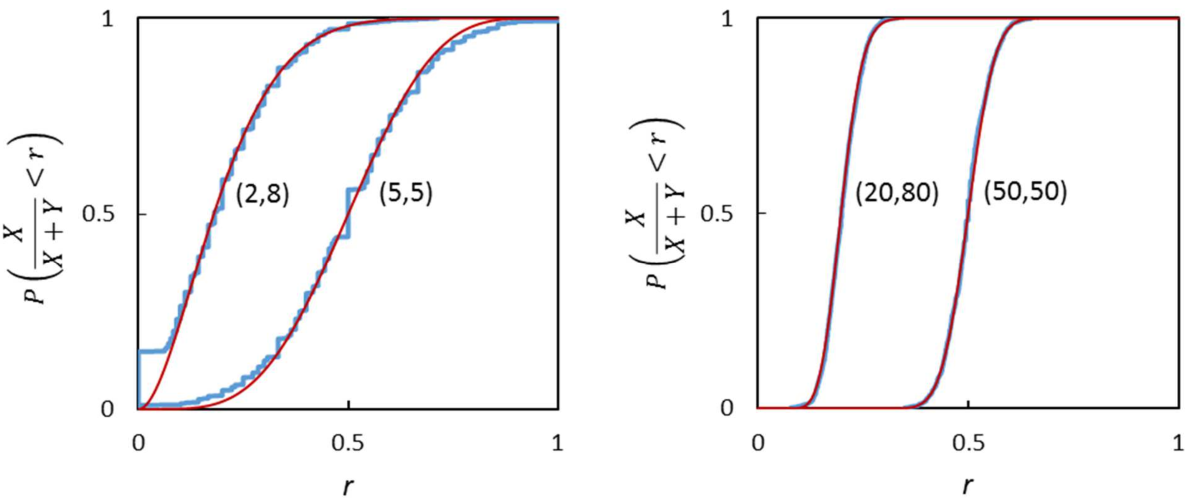

Supplementary Figure 1 | Beta distribution as ratio of Poisson distributions. Agreement between continuous beta distributions (red lines) and discrete distributions of X/(X+Y) (blue lines) for (E(X), E(Y)) equal to (2,8), (5,5), (20,80) and (50,50), where X and Y are Poisson distributed variables. Cumulative distributions are shown in each case. The cumulative distribution function for the ratio of Poisson-distributed variables was generated from 1000 Poisson-distributed (X, Y) pairs.

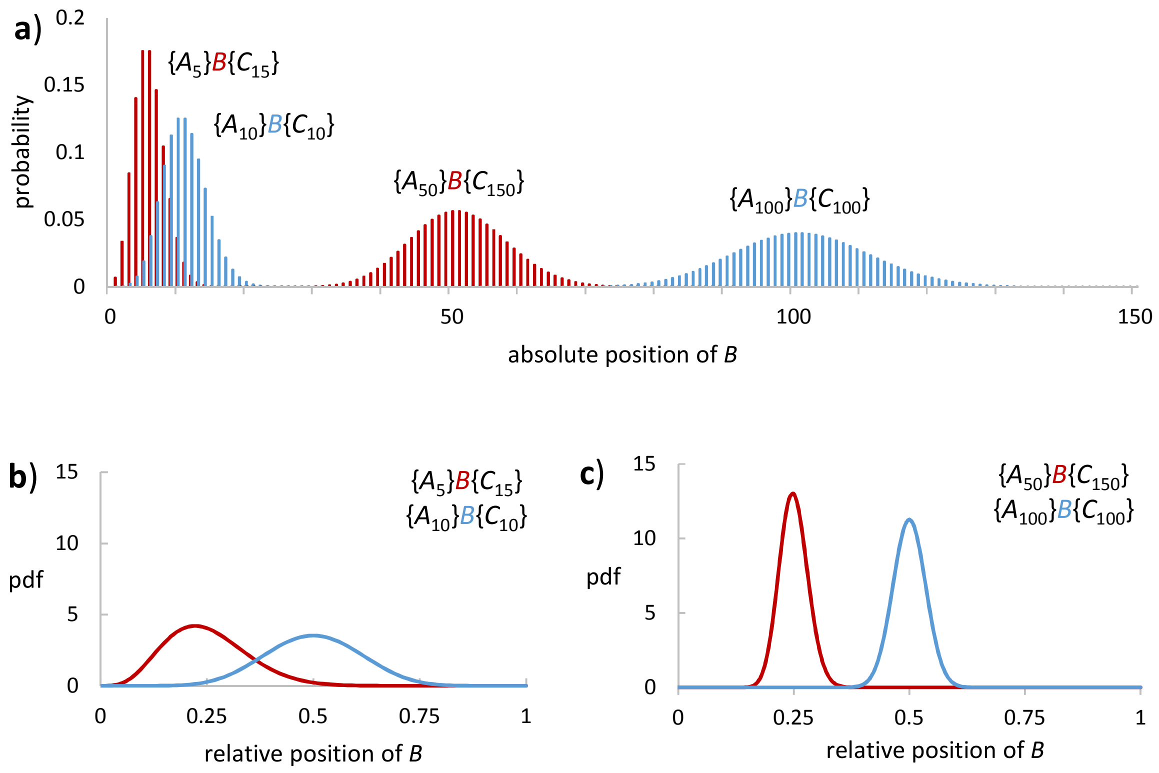

Supplementary Figure 2 | Single monomer insertion. Probability distribution function for the absolute (**a**) and relative (**b**, **c**) locations of the single inserted monomer B in $\{A_5\}B\{C_{15}\}$, $\{A_{10}\}B\{C_{10}\}$, $\{A_{50}\}B\{C_{150}\}$, and $\{A_{100}\}B\{C_{100}\}$ copolymers with Poisson-distributed A and C blocks, illustrating the effect of total average degree of polymerization (21 or 201) and relative location of B (25% or 50%) on the location distribution. The relative location of B in an $\{A_n\}B\{C_n\}$block copolymer is approximated by the Beta(n,m) distribution, while its absolute location is a Poisson(n) distribution, displaced by 1 unit (absolute location = X + 1, where X ~ Poisson(n)).

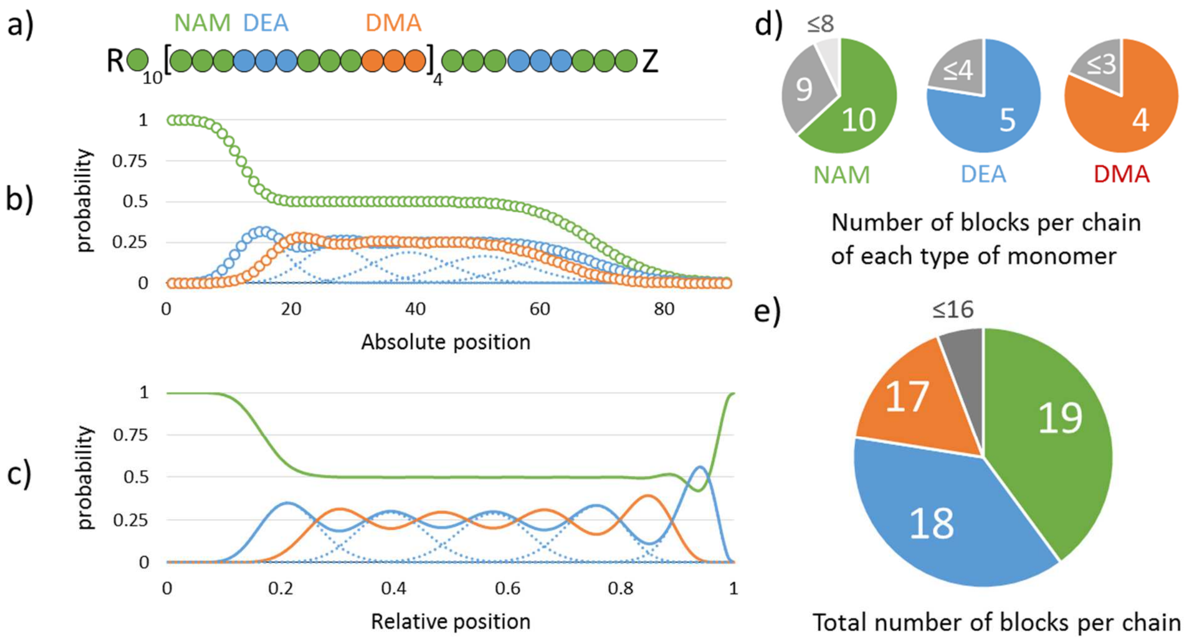

Supplementary Figure 3 | Icosablock copolymer. Icosablock copolymer prepared by sequential RAFT polymerizations of 4-acryloyl morpholine (NAM), *N*,*N*-diethyl acrylamide (DEA) and *N*,*N*-dimethyl acrylamide (DMA).[1] Although the copolymer was prepared by 20 consecutive polymerizations, the first and second blocks are of identical composition, and are counted as a single block for the purposes of the structural analysis. **a**) Reported structure, containing an initial block of NAM_{10} followed by repeated NAM_3-DEA_3-NAM_3-DMA_3 sequences. **b** and **c**) Probability of finding each type of monomer as a function of absolute position (**b**) or relative position (**c**) along polymer chain. Dotted lines represent the distributions of each of the 5 DEA blocks. **d** and **e**) Pie charts showing the fraction of chains which contain the required number of blocks of each type of monomer (**d**) and the fraction of chains which contain all 19, 18, 17 or less than 16 blocks in total (counting the initial consecutive blocks of NAM as a single block).

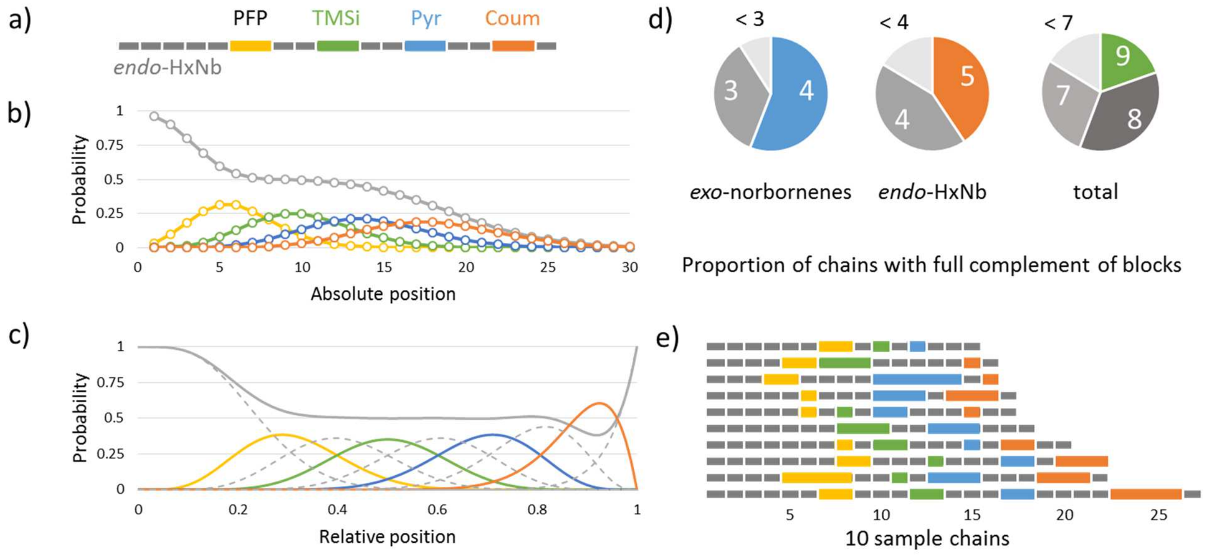

Supplementary Figure 4 | ROMP multisite copolymer. Nonablock copolymer prepared by sequential additions of pentafluorophenyl *exo*-5-norbornene-2-carboxylate (PFP), (1-pyrenyl)methyl *exo*-5-norbornene-2-carboxylate (Pyr), (trimethylsilanyl)methyl *exo*-5-norbornene-2-carboxylate (TMSi), and 7-coumarinyl *exo*-5-norbornene-2-carboxylate (Coum) to a polymerization of *N*-hexyl *endo*-norbornene-5,6-dicarboximide (*endo*-HxNb).[2] **a**) Reported structure, containing blocks of PFP, Pyr, TMSi and Coum (2 units each) separated by *endo*-HxNb spacers (2 units each). An initial block of 5 units *endo*-HxNb and a terminal block of 1 unit *endo*-HxNb complete the structure. **b** and **c**) Probability of finding each type of monomer as a function of absolute position (**b**) or relative position (**c**) along polymer chain. Dotted lines in (**c**) represent the distributions of each of the 5 *endo*-HxNb blocks. The corresponding lines in (**b**) have been omitted for clarity. **d**) Pie charts showing the fraction of chains which contain the required number of functional blocks (*exo*-norbornenes), spacer blocks (*endo*-HxNb) and the total number of blocks. (**e**) A randomly generated sample of 10 chains, illustrating the distribution of structures obtained in the polymerization.

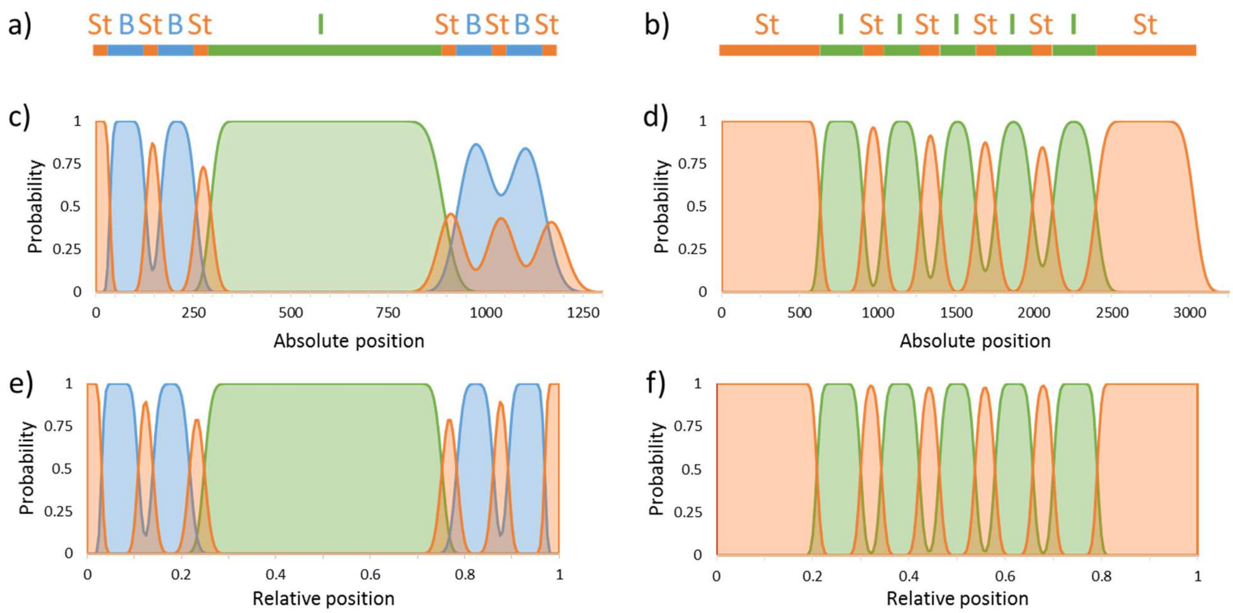

Supplementary Figure 5 | Anionic multiblock copolymers. Undecablock copolymers[3,4] prepared by sequential polymerizations of styrene (St), butadiene (B), or isoprene (I). **a** and **b**) Reported structures $\{St_{37}B_{92}St_{37}B_{92}I_{598}St_{37}B_{92}St_{37}B_{92}\}$[3] (**a**) and $\{St_{633}I_{276}St_{130}I_{231}St_{126}I_{225}St_{126}I_{231}St_{130}I_{276}St_{633}\}$[4] (**b**). **c** and **d**) Probability of finding each type of monomer as a function of absolute position. **e** and **f**) Probability of finding each type of monomer as a function of relative position with respect to the total length of the chain.

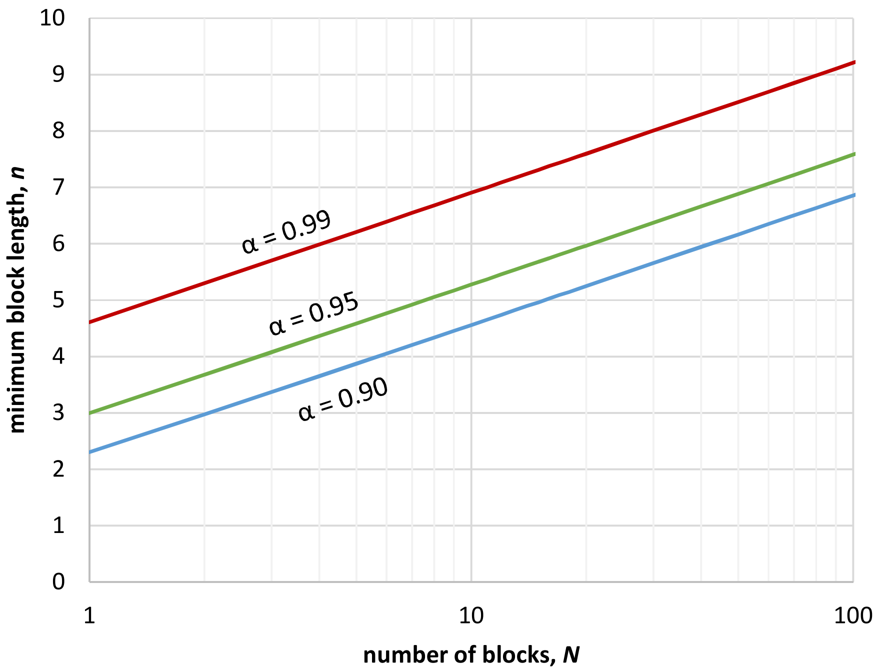

Supplementary Figure 6 | Multiblock copolymer fidelity. Minimum block length, *n*, required to produce an *N*-block copolymer with 90%, 95%, or 99% fidelity assuming Poisson-distributed blocks of equal average length and absence of termination.

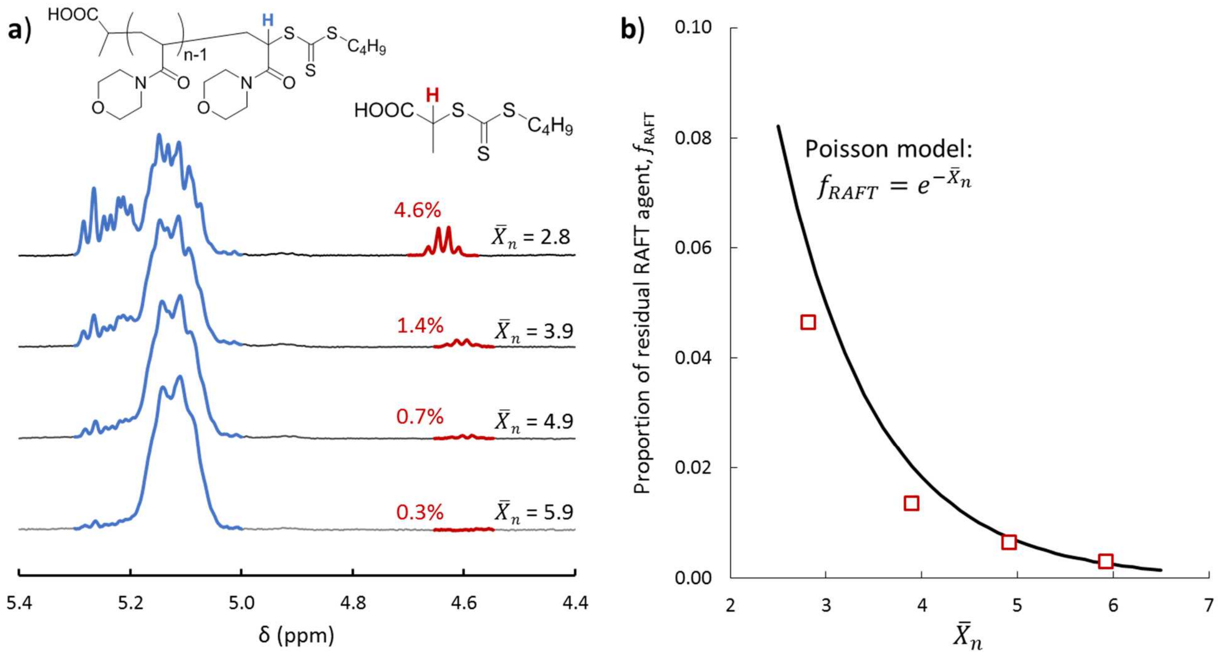

Supplementary Figure 7 | Proportion of polymer with $\bar{X}_n = 0$ in short blocks. a) Partial NMR spectra of oligomers of 4-acryloylmorpholine (NAM) prepared by RAFT polymerization, showing quartet at 4.6 ppm (in red) corresponding to unreacted RAFT agent. The proportion of RAFT agent, f_{RAFT}, calculated by integrating this quartet and the corresponding multiplet due to NAM oligomers at 5.0-5.3 ppm (blue), agrees well with the prediction, assuming a Poisson distribution of chain lengths (**b**).

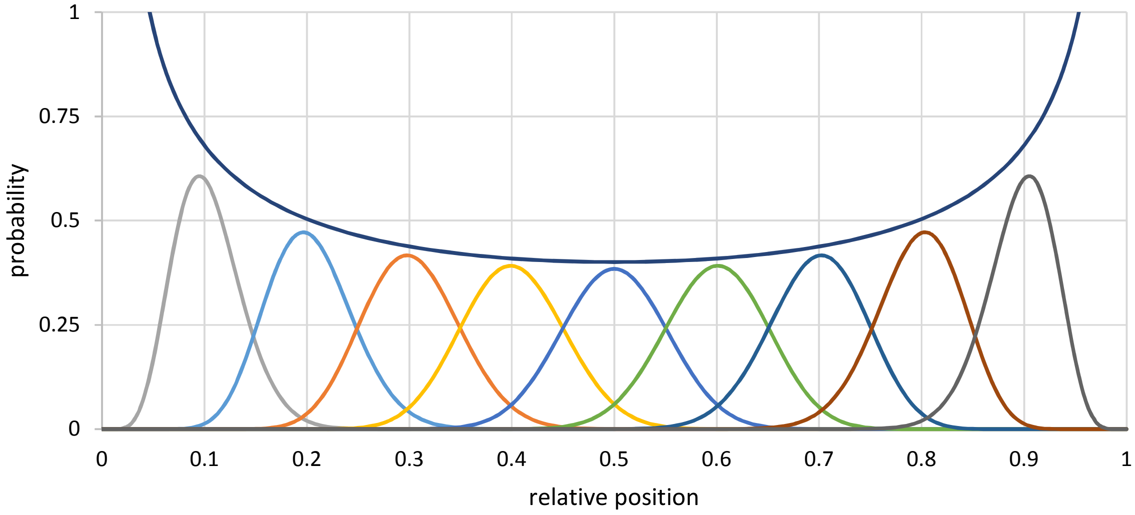

Supplementary Figure 8 | Approximation of maximum probability (See Supplementary Note 1). Distribution of blocks of DP_n 5 in a polymer of DP_n 100 as a function of insertion point. Expected position of blocks varies from 0.1 to 0.9 in increments of 0.1. Approximate maximum probability is also shown (calculated from $5/\sqrt{(\pi H(100r+\frac{1}{2},100(1-r)+\frac{1}{2})}$, where H($a$,$b$) is the harmonic mean of a and b.

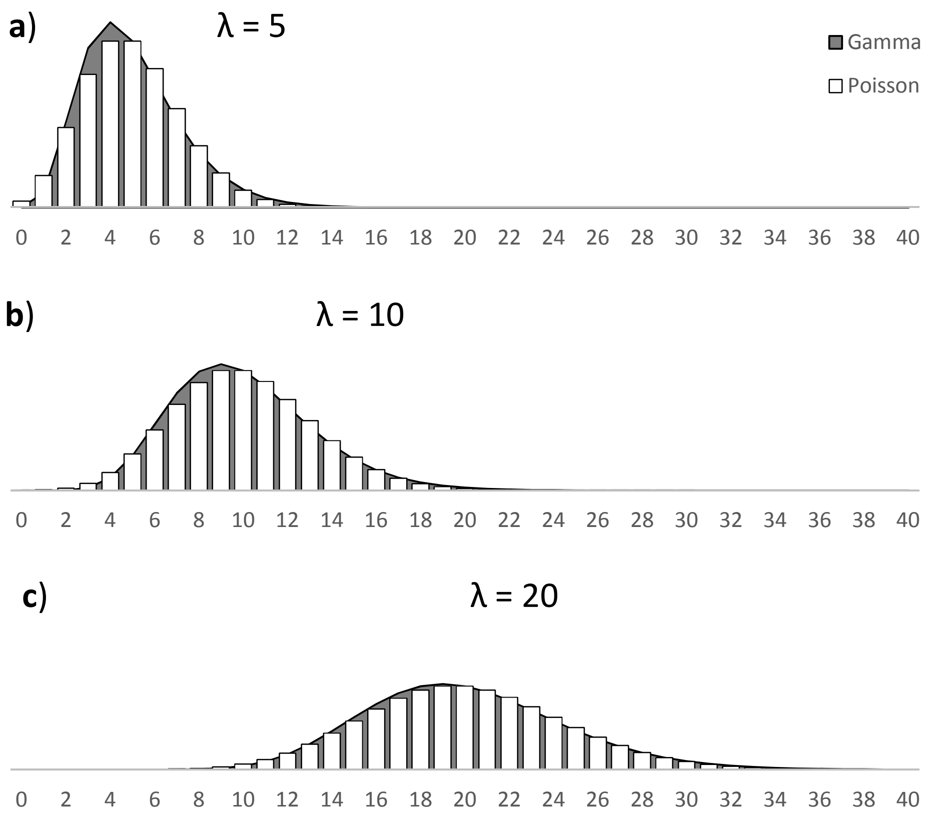

Supplementary Figure 9 | Superposition of Poisson and Gamma distributions (see Supplementary Note 2). Discrete *Poisson(λ)* distribution superimposed on continuous *Gamma(λ,1)* distribution for *λ* equal to 5 (**a**), 10 (**b**) and 20 (**c**).

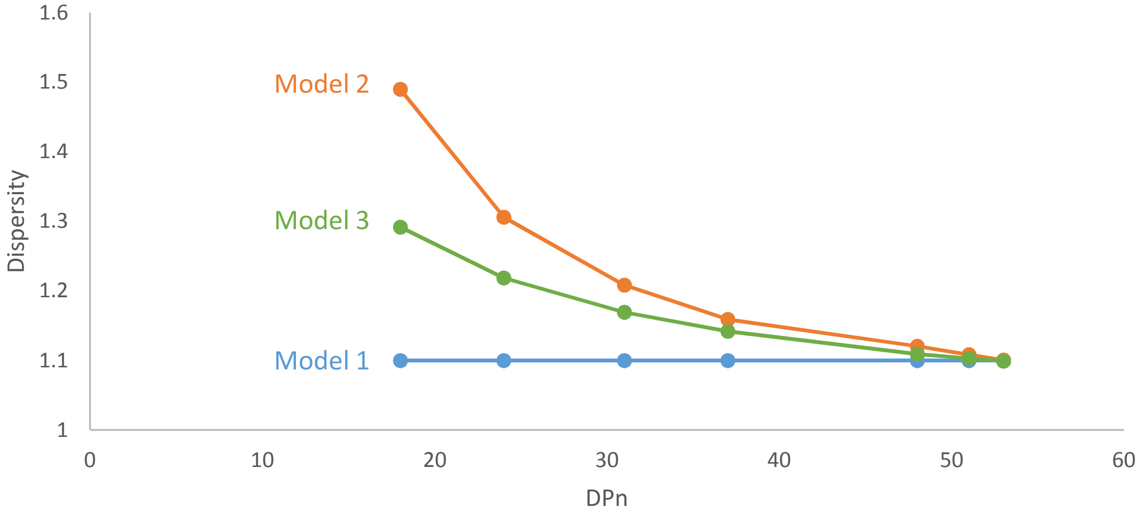

Supplementary Figure 10 | Evolution of cumulative dispersity for the three models considered in Supplementary Note 4. Model 1 (blue): constant cumulative dispersity. Model 2 (orange): constant segment dispersity. Model 3 (green): dispersity equal to $1 + a/DP_n$ (a = 5.25).

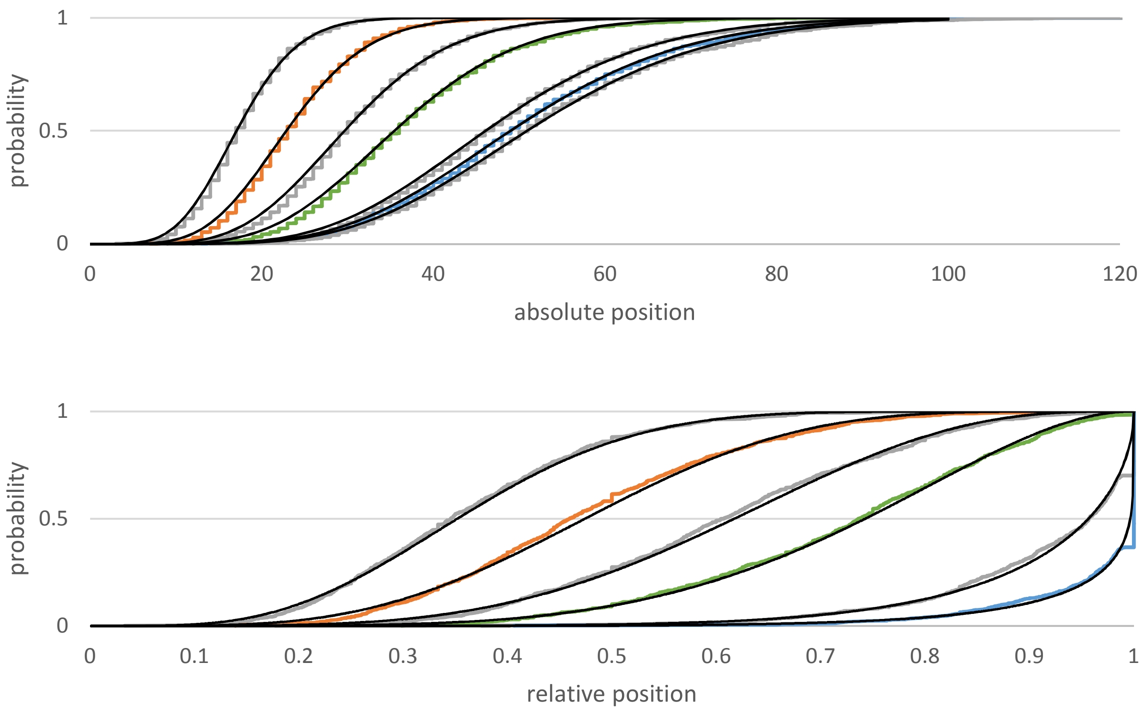

Supplementary Figure 11 | Absolute and relative positions of interfaces between blocks of a styrene-maleimide multiblock copolymer (Supplementary Note 4) with constant cumulative dispersity of 1.10 (model 1). Thick lines represent empirically generated cumulative probability distributions, thin lines negative binomial (absolute) or beta (relative) distributions obtained by matching moments. Colored lines represented blocks containing a functional maleimide (orange: BzMI; green: PrMI; blue: PFPMI). Gray lines represent non-functional polystyrene blocks.

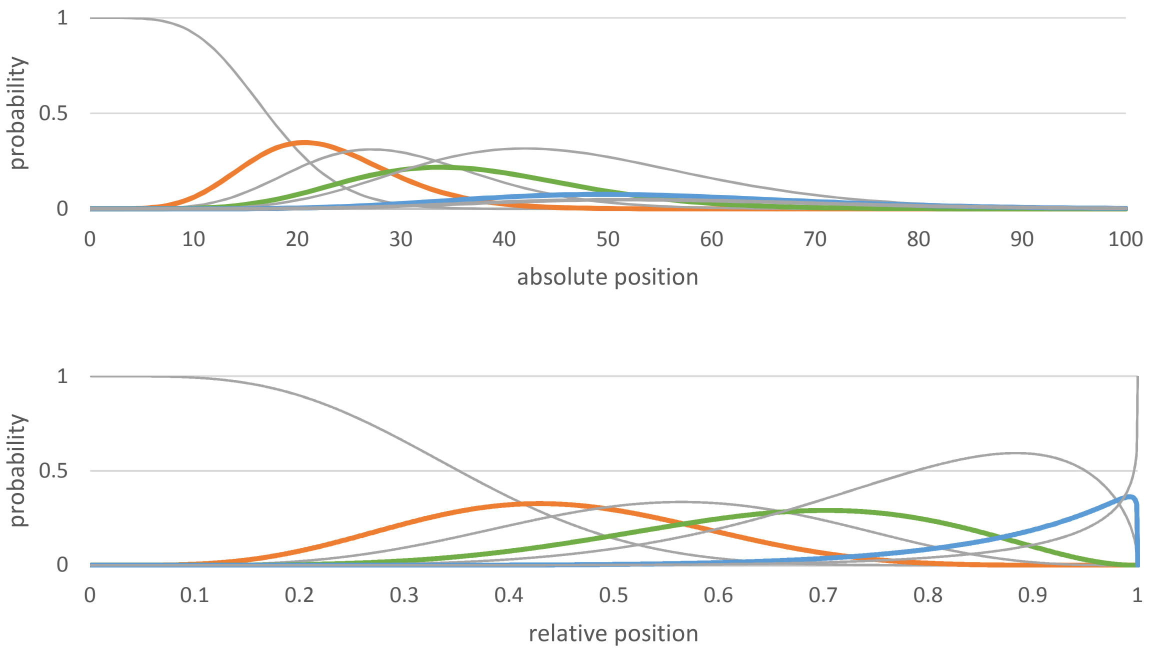

Supplementary Figure 12 | Absolute (top) and relative (bottom) distributions of segments of a multiblock styrene-maleimide copolymer (dispersity model 1, Supplementary Note 4), obtained from the difference between cumulative distributions of Supplementary Fig. 11. Colored lines represented blocks containing a functional maleimide (orange: BzMI; green: PrMI; blue: PFPMI). Gray lines represent non-functional polystyrene blocks.

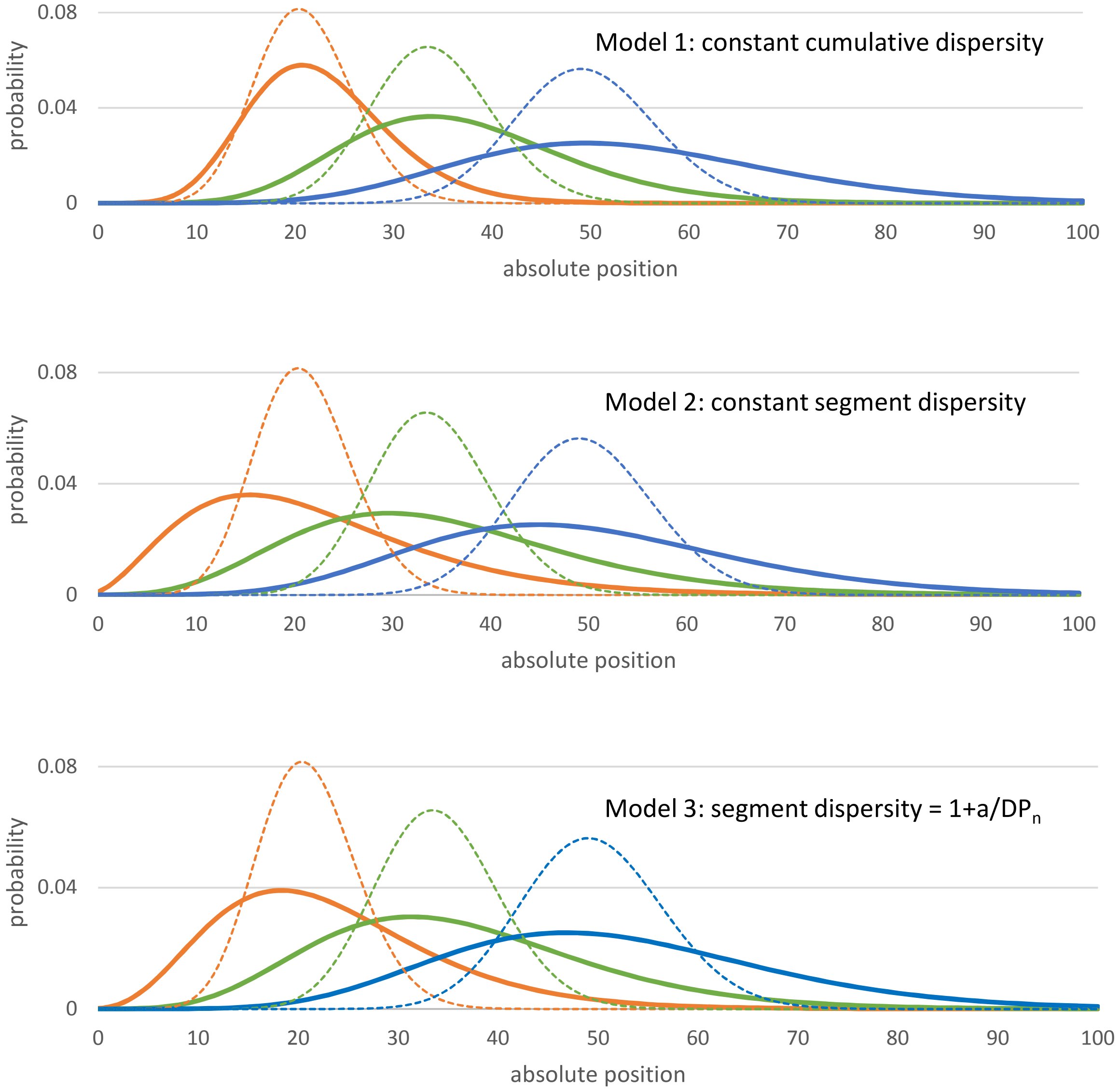

Supplementary Fig. 13 | Absolute distributions of functional maleimide monomers in a multiblock styrene-maleimide copolymer of dispersity 1.10 (Supplementary Note 4), according to the three models of segment dispersity shown in Supplementary Table 3. Dashed lines represent the corresponding distributions assuming Poisson-distributed segments. Colored lines represented blocks containing a functional maleimide (orange: BzMI; green: PrMI; blue: PFPMI). Gray lines represent non-functional polystyrene blocks.

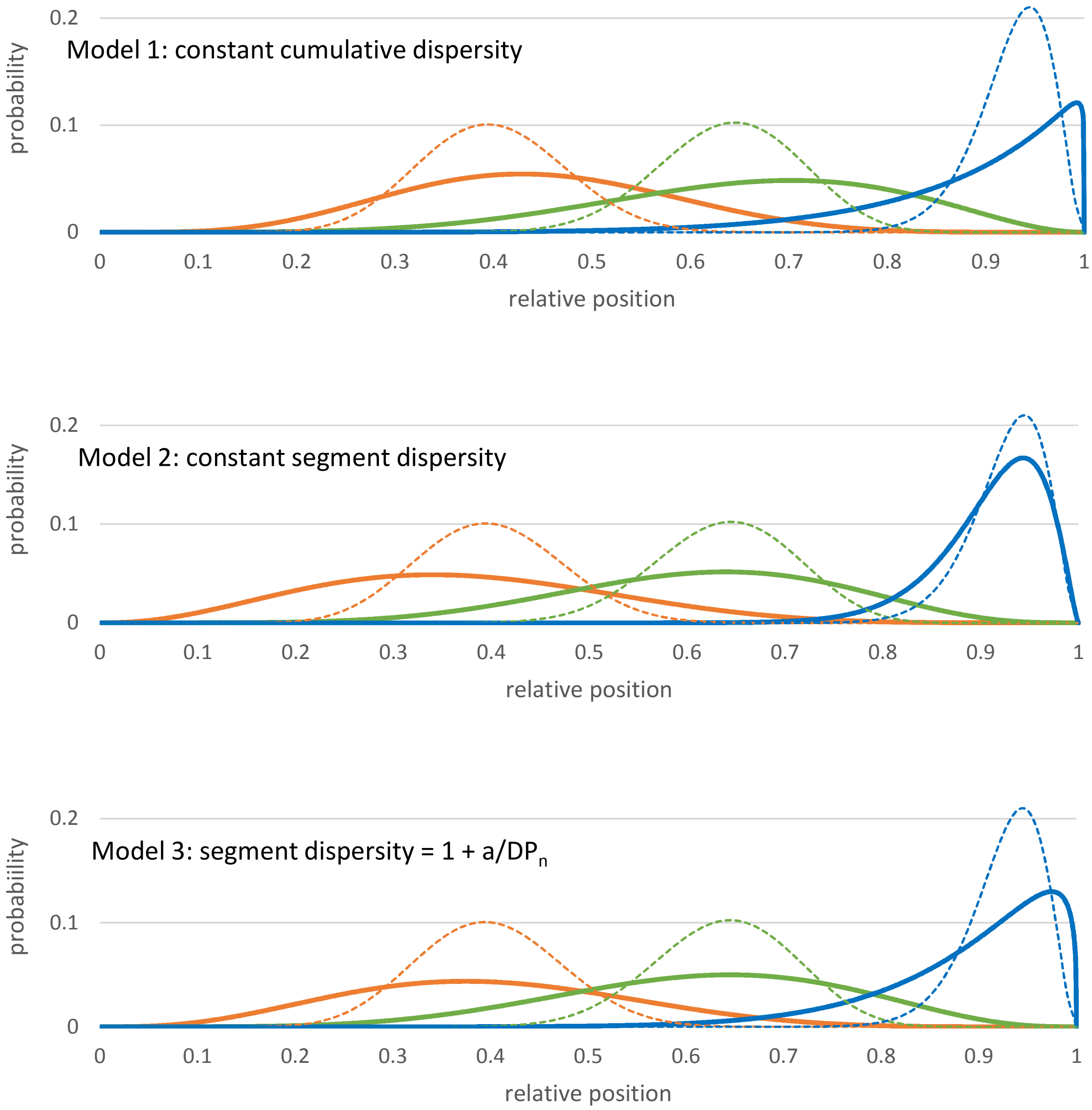

Supplementary Figure 14 | Relative distributions of functional maleimide monomers in a multiblock styrene-maleimide copolymer of dispersity 1.10 (Supplementary Note 4), according to the three models of segment dispersity shown in Supplementary Table 3. Dashed lines represent the corresponding distributions assuming Poisson-distributed segments. Colored lines represented blocks containing a functional maleimide (orange: BzMI; green: PrMI; blue: PFPMI). Gray lines represent non-functional polystyrene blocks.

Supplementary Table 1 | Special functions used in Table 1.

Incomplete gamma function:	$\Gamma(s,x) = \int_x^\infty t^x e^{-t} dt$
Beta function:	$B(a,b) = \int_0^1 t^{a-1}(1-t)^{b-1} dt$
Regularized incomplete beta function:	$I_x(a,b) = \frac{\int_0^x t^{a-1}(1-t)^{b-1} dt}{B(a,b)}$
Binomial coefficient:	$\binom{n}{k} = \frac{n!}{k!\,(n-k)!}$
Harmonic mean:	$H(a,b) = \left(\frac{a^{-1}+b^{-1}}{2}\right)^{-1}$

Supplementary Table 2 | 'Conventional' vs 'ultraprecise' maleimide insertion. Expected position and standard deviation (in parentheses) of Bz-MI, Pr-MI and PFP-MI units in polystyrene-based copolymers of total $\bar{X}_n$ = 53, prepared by sequential maleimide additions (conventional) or alternating maleimide and styrene additions (ultraprecise) techniques,[5] assuming Poisson-distributed segments.

	**Bz-MI**		**Pr-MI**		**PFP-MI**	
	absolute	relative	absolute	relative	absolute	relative
conventional	22.0 (4.9)	0.40 (0.073)	35.0 (6.1)	0.64 (0.072)	50.5 (7.1)	0.93 (0.038)
ultraprecise	19.5 (4.4)	0.35 (0.066)	32.5 (5.7)	0.59 (0.068)	49.5 (7.0)	0.91 0.042)

Supplementary Table 3 | Segment dispersities for a multiblock copolymer with overall dispersity of 1.10, and parameters (*p*, *r*) for negative binomial distributions with the same number average and dispersity.

**Block**<br>**Monomer**<br>**DP_n**	**1**<br>**St**<br>**18**	**2**<br>**St/BzMI**<br>**6**	**3**<br>**St**<br>**7**	**4**<br>**St/PrMI**<br>**6**	**5**<br>**St**<br>**11**	**6**<br>**St/PFPMI**<br>**3**	**7**<br>**St**<br>**2**	**Total**<br><br>**53**
Đ (Poisson)	1.06	1.17	1.14	1.17	1.09	1.33	1.50	1.02
Đ (Model 1)	1.10	1.70	1.79	2.13	1.77	4.30	6.20	1.10
p	0.444	0.762	0.818	0.853	0.882	0.899	0.904	
r	22.5	1.88	1.56	1.03	1.47	0.337	0.213	
Đ (Model 2)	1.49	1.49	1.49	1.49	1.49	1.49	1.50	1.10
p	0.887	0.660	0.708	0.660	0.814	0.320	0.111	
r	2.30	3.09	2.88	3.09	2.31	6.38	16	
Đ (Model 3)	1.29	1.88	1.75	1.88	1.48	2.75	3.63	1.10
p	0.810	0.810	0.810	0.810	0.810	0.810	0.810	
r	4.24	1.41	1.65	1.41	2.59	0.706	0.471	

Supplementary Note 1. Derivation of results for the location of Poisson-distributed blocks in a Poisson-distributed multiblock copolymer.

Absolute location.

In a multiblock copolymer in which all block lengths are Poisson distributed, the probability that the kth monomer, M_k, will be part of the nth block is given by the difference between two Poisson cumulative distribution functions:

$$P(M_k \text{ in block } n) = P(L_{n-1} \leq k-1) - P(L_n \leq k-1) = \frac{\Gamma(k, L_{n-1}) - \Gamma(k, L_n)}{\Gamma(k)} \quad (1)$$

where L_n and L_{n-1} represent the total average lengths of the first n and n-1 blocks respectively, and $\Gamma(s,x)$ is the upper incomplete gamma function, defined in Supplementary Table 1.

Total probability of finding a monomer from a given block. If the probability of finding a monomer from the nth block in the ith position of the polymer is given by $P(M_i$ in block $n)$, the sum of the probabilities for all i is equal to the expected number of monomers in the block, $L_n - L_{n-1}$.

$$\sum_{i=1}^{\infty} P(M_i \text{ in block } n) = L_n - L_{n-1} \quad (2)$$

Proof. The sum of the probabilities is given by

$$\sum_{i=1}^{\infty} P(M_i \text{ in block } n) = \sum_{i=1}^{\infty} \frac{\Gamma(i, L_{n-1}) - \Gamma(i, L_n)}{\Gamma(i)} \quad (3)$$

Using an alternative formulation for the cumulative distribution function of the Poisson distribution, this can be expressed:

$$\sum_{i=1}^{\infty} P(M_i \text{ in block } n) = \sum_{i=1}^{\infty} \sum_{j=0}^{i-1} \frac{e^{-L_{n-1}} L_{n-1}^j - e^{-L_n} L_n^j}{j!} \quad (4)$$

As $\sum_{j=0}^{\infty} \frac{x^j}{j!}$ is the Taylor series expansion of e^x, the sum of probabilities can be expressed as

$$\sum_{i=1}^{\infty} P(M_i \text{ in block } n) = \sum_{i=1}^{\infty}\left(e^{-L_{n-1}}\left(e^{L_{n-1}} - \sum_{j=i}^{\infty}\frac{L_{n-1}^j}{j!}\right) - e^{-L_n}\left(e^{L_n} - \sum_{j=i}^{\infty}\frac{L_n^j}{j!}\right)\right)$$

$$= \sum_{i=1}^{\infty}\left(e^{-L_n}\left(\sum_{j=i}^{\infty}\frac{L_n^j}{j!}\right) - e^{-L_{n-1}}\left(\sum_{j=i}^{\infty}\frac{L_{n-1}^j}{j!}\right)\right)$$

(5)

Expanding the double sum gives

$$\sum_{i=1}^{\infty} P(M_i \text{ in block } n)$$
$$= e^{-L_n}\left(\frac{L_n^1}{1!} + \frac{2L_n^2}{2!} + \frac{3L_n^3}{3!} + \cdots\right) - e^{-L_{n-1}}\left(\frac{L_{n-1}^1}{1!} + \frac{2L_{n-1}^2}{2!} + \frac{3L_{n-1}^3}{3!} + \cdots\right)$$

(6)

Which can be rewritten as

$$\sum_{i=1}^{\infty} P(M_i \text{ in block } n) = \sum_{i=1}^{\infty}\left(e^{-L_n}\frac{iL_n^i}{i!} - e^{-L_{n-1}}\frac{iL_{n-1}^i}{i!}\right) = \sum_{i=1}^{\infty}\left(e^{-L_n}\frac{L_n^i}{(i-1)!} - e^{-L_{n-1}}\frac{L_{n-1}^i}{(i-1)!}\right)$$

(7)

This in turn contains the Taylor series expansion of xe^x, $\sum_{i=1}^{\infty}\frac{x^i}{(i-1)!}$, and can thus be simplified to

$$\sum_{i=1}^{\infty} P(M_i \text{ in block } n) = e^{-L_n}L_n e^{L_n} - e^{-L_{n-1}}L_{n-1}e^{L_{n-1}} = L_n - L_{n-1}$$

(2)

Expected location of a given block. The expected value of the location of the nth block is given by:

$$E(X) = 1 + \frac{L_n + L_{n-1}}{2}$$

(8)

Proof. The expected location of the nth block is obtained from the sum of all the possible locations, weighted by the probability of finding the block in that location:

$$E(X) = \frac{\sum_{i=1}^{\infty} i\,\frac{\Gamma(i,L_{n-1}) - \Gamma(i,L_n)}{\Gamma(i)}}{L_n - L_{n-1}} = \frac{1}{L_n - L_{n-1}}\sum_{i=1}^{\infty} i \sum_{j=0}^{i-1}\frac{e^{-L_{n-1}}L_{n-1}^j - e^{-L_n}L_n^j}{j!}$$

(9)

Following the same procedure as for the sum of probabilities, this can be simplified to

$$E(X) = \frac{1}{L_n - L_{n-1}} \sum_{i=1}^{\infty} \left(ie^{-L_n} \left(\sum_{j=i}^{\infty} \frac{L_n^j}{j!} \right) - ie^{-L_{n-1}} \left(\sum_{j=i}^{\infty} \frac{L_{n-1}^j}{j!} \right) \right)$$
$$= \frac{e^{-L_n}}{L_n - L_{n-1}} \left(\frac{L_n^1}{1!} + \frac{(1+2)L_n^2}{2!} + \frac{(1+2+3)L_n^3}{3!} + \cdots \right)$$
$$- \frac{e^{-L_{n-1}}}{L_n - L_{n-1}} \left(\frac{L_{n-1}^1}{1!} + \frac{(1+2)L_{n-1}^2}{2!} + \frac{(1+2+3)L_{n-1}^3}{3!} + \cdots \right)$$
$$= \frac{1}{L_n - L_{n-1}} \sum_{i=1}^{\infty} \left(e^{-L_n} \frac{i(i+1)L_n^i}{2.i!} - e^{-L_{n-1}} \frac{i(i+1)L_{n-1}^i}{2.i!} \right)$$

(10)

The sum $\sum_{i=1}^{\infty} \frac{i(i+1)x^i}{2.i!}$ is the Taylor series expansion of $\left(x + \frac{x^2}{2}\right).e^x$. Substituting this for the infinite sums gives

$$E(X) = \frac{\left(L_n + \frac{L_n^2}{2}\right) - \left(L_{n-1} + \frac{L_{n-1}^2}{2}\right)}{L_n - L_{n-1}} = 1 + \frac{L_n + L_{n-1}}{2}$$

(8)

Variance of the location. The variance of the location of the *n*th block is given by

$$Var(X) = \frac{(L_n - L_{n-1})^2 + 6(L_n + L_{n-1})}{12} = \frac{(L_n - L_{n-1})^2}{12} + \frac{L_n + L_{n-1}}{2}$$

(11)

The standard deviation is obtained by taking the square root of the variance.

$$\sigma(X) = \sqrt{\frac{(L_n - L_{n-1})^2}{12} + E(X) - 1}$$

(12)

For comparison, a discrete uniform distribution, $\mathcal{U}\{L_{n-1} + 1, L_n\}$, in which there is equal probability of finding the desired monomer at any point in the range [L_{n-1}+1, L_n], has the following expected value and standard deviation:

$$E(X) = \frac{L_n + L_{n-1} + 1}{2} \quad (X \sim \mathcal{U}\{L_{n-1} + 1, L_n\})$$

(13)

$$\sigma(X) = \sqrt{\frac{(L_n - L_{n-1})^2 - 1}{12}}$$

(14)

Proof. The variance of X is obtained from the formula $Var(X) = E(X^2)-[E(X)]^2$.

$$E(X^2) = \frac{1}{L_n - L_{n-1}} \sum_{i=1}^{\infty} \left(i^2 e^{-L_n} \left(\sum_{j=i}^{\infty} \frac{L_n^j}{j!} \right) - i^2 e^{-L_{n-1}} \left(\sum_{j=i}^{\infty} \frac{L_{n-1}^j}{j!} \right) \right)$$

$$= \frac{e^{-L_n}}{L_n - L_{n-1}} \left(\frac{L_n^1}{1!} + \frac{(1+4)L_n^2}{2!} + \frac{(1+4+9)L_n^3}{3!} + \cdots \right) - \frac{e^{-L_{n-1}}}{L_n - L_{n-1}} \left(\frac{L_{n-1}^1}{1!} + \frac{(1+4)L_{n-1}^2}{2!} + \frac{(1+4+9)L_{n-1}^3}{3!} + \cdots \right)$$

$$= \frac{1}{L_n - L_{n-1}} \sum_{i=1}^{\infty} \left(e^{-L_n} \frac{i(i+1)(2i+1)L_n^i}{6.\,i!} - e^{-L_{n-1}} \frac{i(i+1)(2i+1)L_{n-1}^i}{6.\,i!} \right)$$

$$= e^{-L_n} \left(\frac{6L_n + 9{L_n}^2 + 2{L_n}^3}{6(L_n - L_{n-1})} \right) e^{L_n} - e^{-L_{n-1}} \left(\frac{6L_{n-1} + 9{L_{n-1}}^2 + 2{L_{n-1}}^3}{6(L_n - L_{n-1})} \right) e^{L_{n-1}}$$

$$= \frac{6 + 9(L_n + L_{n-1}) + 2(L_n^2 + L_n L_{n-1} + L_{n-1}^2)}{6}$$

(15)

The variance is obtained by subtracting the square of the expected value:

$$Var(X) = E(X^2) - [E(X)]^2 = \frac{6 + 9(L_n + L_{n-1}) + 2(L_n^2 + L_n L_{n-1} + L_{n-1}^2)}{6} - \left(1 + \frac{L_n + L_{n-1}}{2}\right)^2$$

$$= \frac{6 + 9(L_n + L_{n-1}) + 2(L_n^2 + L_n L_{n-1} + L_{n-1}^2)}{6} - \left(1 + L_n + L_{n-1} + \frac{L_n^2 + 2L_n L_{n-1} + L_{n-1}^2}{4}\right)$$

$$= \frac{6(L_n + L_{n-1}) + (L_n^2 - 2L_n L_{n-1} + L_{n-1}^2)}{12}$$

$$Var(X) = \frac{(L_n - L_{n-1})^2 + 6(L_n + L_{n-1})}{12} = \frac{(L_n - L_{n-1})^2}{12} + \frac{L_n + L_{n-1}}{2}$$

(11)

Location with maximum probability (mode). The position, k_{max}, with maximum probability of finding a monomer from the nth block is:

$$k_{max} = 1 + \left\lfloor \frac{L_n - L_{n-1}}{\ln(L_n) - \ln(L_{n-1})} \right\rfloor \tag{16}$$

where $\lfloor x \rfloor$ represents the integer part of *x*.

If $L_n - L_{n-1} << L_n$, this can be approximated by the integer part of the expected location of the *n*th block:

$$k_{max} \approx 1 + \left\lfloor \frac{L_n + L_{n-1}}{2} \right\rfloor = \lfloor E(X) \rfloor \tag{17}$$

Proof. The change in probability going from position *k* to position *k*+1 is given by

$$P(M_k \text{ in block } n) - P(M_{k+1} \text{ in block } n) = \sum_{i=0}^{k-1} \frac{e^{-L_{n-1}} L_{n-1}^i - e^{-L_n} L_n^i}{i!} - \sum_{i=0}^{k} \frac{e^{-L_{n-1}} L_{n-1}^i - e^{-L_n} L_n^i}{i!}$$
$$= \frac{e^{-L_n} L_n^k - e^{-L_{n-1}} L_{n-1}^k}{k!} \tag{18}$$

The maximum probability occurs when the probability in position *k* is greater than the probabilities in both position *k*-1 and position *k*+1. That is,

$$\frac{e^{-L_n} L_n^{k-1} - e^{-L_{n-1}} L_{n-1}^{k-1}}{(k-1)!} < 0 \; and \; \frac{e^{-L_n} L_n^k - e^{-L_{n-1}} L_{n-1}^k}{k!} > 0 \tag{19}$$

These conditions are fulfilled when $k - 1 < \frac{L_n - L_{n-1}}{\ln(L_n) - \ln(L_{n-1})} < k$.

As *k* must be an integer,

$$k_{max} = 1 + \left\lfloor \frac{L_n - L_{n-1}}{\ln(L_n) - \ln(L_{n-1})} \right\rfloor \tag{16}$$

where $\lfloor x \rfloor$ represents the integer part of *x*.

If the difference between L_n and L_{n-1} is small relative to L_n, $ln(L_n)$-$ln(L_{n-1})$ can be approximated by $(L_n$-$L_{n-1})$ times the derivative of *ln(x)* evaluated midway between L_n and L_{n-1}. The resulting value is the integer part of the expected value of the location of the block.

$$k_{max} \approx 1 + \left\lfloor \frac{L_n - L_{n-1}}{(L_n - L_{n-1}) \left[\frac{d \ln x}{dx}\right]_{x=\frac{L_n + L_{n-1}}{2}}} \right\rfloor$$

$$k_{max} \approx 1 + \left\lfloor \frac{L_n + L_{n-1}}{2} \right\rfloor = \lfloor E(X) \rfloor$$

(17)

Approximate maximum probability of finding a given monomer at any position. The approximate maximum probability of finding the desired monomer is

$$P(M_{k_{max}} \text{ in block } n) \approx \frac{L_n - L_{n-1}}{\sqrt{2\pi(k_{max} - 1)}}$$

(20)

This probability increases with the expected block length (L_n – L_{n-1}) and decreases with the square root of k_{max}.

Proof. The approximate maximum probability of finding the desired monomer is obtained by evaluating the probability at k_{max}, using $(L_{n-1} - L_n) \frac{\partial \Gamma(k_{max}, x)}{\partial x}$ evaluated at $x = k_{max}$-1 (roughly halfway between L_n and L_{n-1}) as an approximation for $\Gamma(k_{max}, L_{n-1}) - \Gamma(k_{max}, L_n)$.

Stirling's approximation for $x!$ is used to simplify the resulting expression.

$$P(M_{k_{max}} \text{ in block } n) = \frac{\Gamma(k_{max}, L_{n-1}) - \Gamma(k_{max}, L_n)}{\Gamma(k_{max})} \approx \frac{L_{n-1} - L_n}{\Gamma(k_{max})} \left[\frac{\partial \Gamma(k_{max}, x)}{\partial x}\right]_{x=k_{max}-1}$$

$$P\left(M_{k_{max}} \text{ in block } n\right) \approx \frac{L_n - L_{n-1}}{(k_{max} - 1)!} \frac{(k_{max} - 1)^{k_{max}-1}}{e^{k_{max}-1}} \approx \frac{L_n - L_{n-1}}{\sqrt{2\pi(k_{max} - 1)}}$$

(20)

Relative location.

In a multiblock copolymer in which the lengths of all blocks are Poisson-distributed, the interface between the nth and $(n+1)^{th}$ blocks is approximately $Beta(L_n, L_{total} - L_n)$ distributed, where L_n represents the sum of the average lengths of the first n blocks, and L_{total} is the total average length of the copolymer.

A given relative location, r, is part of the nth block if the interface between the $(n-1)^{th}$ and n^{th} blocks is less than r, and the the interface between the nth and $(n+1)^{th}$ blocks is greater than r. The probability that r is part of the nth block is given by the difference between two cumulative beta distributions.

$$P(r \text{ part of } n^{\text{th}}\text{block}) = I_r(L_{n-1}, L_{total} - L_{n-1}) - I_r(L_n, L_{total} - L_n) \tag{21}$$

where $I_x(a,b)$ is the regularized incomplete beta function, defined in Supplementary Table 1.

Total probability of finding a monomer from a given block.

The total probability that a location selected at random will correspond to the nth block can be obtained by integrating from $r = 0$ to $r = 1$. For integer values of L_{n-1}, L_n and L_{total}, the integral is given by:

$$P(\text{any } r \text{ part of } n^{\text{th}}\text{block}) = \int_0^1 \big(I_r(L_{n-1}, L_{total} - L_{n-1}) - I_r(L_n, L_{total} - L_n)\big)dr = \frac{L_n - L_{n-1}}{L_{total}} \tag{22}$$

This corresponds to the fraction of the copolymer that is composed of the nth block.

Proof. The proof is by induction:

Let $L_{n-1} = a$, $L_{total} - L_n = b$.

Assume that, for a block of expected length k, the total probability is given by $k/(a+b+k)$.

This is true for $k = 1$:

$$P(\text{any } r \text{ part of } n^{\text{th}}\text{block}) = \int_0^1 \big(I_r(a, b+1) - I_r(a+1, b)\big)dr$$

$$= \int_0^1 \left(I_r(a,b) + \frac{r^a(1-r)^b}{bB(a,b)} - \left(I_r(a,b) - \frac{r^a(1-r)^b}{aB(a,b)}\right)\right)dr = \int_0^1 \frac{r^a(1-r)^b}{B(a,b)}\left(\frac{1}{a} + \frac{1}{b}\right)dr$$

$$= \frac{B(a+1, b+1)}{B(a,b)}\left(\frac{a+b}{ab}\right)$$

$$B(a+1, b+1) = B(a, b+1)\frac{a}{a+b+1} = B(a,b)\frac{ab}{(a+b+1)(a+b)}$$

$$P(\text{any } r \text{ part of } n^{\text{th}}\text{block}) = \frac{ab}{(a+b+1)(a+b)}\left(\frac{a+b}{ab}\right) = \frac{1}{a+b+1} \tag{23}$$

And also for $k = k + 1$:

$$
\begin{aligned}
&P\big(\text{any } r \text{ part of } n^{\text{th}}\text{block}\big) \\
&= \int_0^1 \big(I_r(a, b+k+1) - I_r(a+k+1, b)\big)dr \\
&= \int_0^1 \left(I_r(a, b+k) + \frac{r^a(1-r)^{b+k}}{(b+k)B(a, b+k)} - \left(I_r(a+k, b) - \frac{r^{a+k}(1-r)^b}{(a+k)B(a+k, b)}\right)\right)dr \\
&= \int_0^1 \big(I_r(a, b+k) - I_r(a+k, b)\big)dr \\
&+ \int_0^1 \left(\frac{r^{a+k}(1-r)^b}{(a+k)B(a+k, b)} + \frac{r^a(1-r)^{b+k}}{(b+k)B(a, b+k)}\right)dr \\
&= \frac{k}{a+b+k} + \frac{B(a+k+1, b+1)}{(a+k)B(a+k, b)} + \frac{B(a+1, b+k+1)}{(b+k)B(a, b+k)} \\
&= \frac{k}{a+b+k} + \frac{B(a+k, b)(a+k)b}{(a+k)B(a+k, b)(a+b+k+1)(a+b+k)} \\
&+ \frac{B(a, b+k)a(b+k)}{(b+k)B(a, b+k)(a+b+k+1)(a+b+k)} \\
&= \frac{k}{a+b+k} + \frac{a+b}{(a+b+k+1)(a+b+k)} = \frac{k+1}{(a+b+k+1)}
\end{aligned}
$$

(24)

Thus the total probability that a randomly selected relative location will form part of a block of length (L_n–L_{n-1}) is equal to

$$
P\big(\text{any } r \text{ part of } n^{\text{th}}\text{block}\big) = \frac{k}{a+b+k} = \frac{L_n - L_{n-1}}{L_{total}}
$$

(22)

Expected relative location. The expected relative location, *E(R)*, of a block is given by

$$
E(R) = \frac{L_n + L_{n-1} + 1}{2(L_{total} + 1)}
$$

(25)

for all integer values of L_{n-1}, L_n and L_{total}..

Proof. Proof is by induction.

$$
\begin{aligned}
E(R) &= \frac{\int_0^1 r\big(I_r(L_{n-1}, L_{total} - L_{n-1}) - I_r(L_n, L_{total} - L_n)\big)dr}{\int_0^1\big(I_r(L_{n-1}, L_{total} - L_{n-1}) - I_r(L_n, L_{total} - L_n)\big)dr} \\
&= \frac{L_{total}}{L_n - L_{n-1}} \int_0^1 r\big(I_r(L_{n-1}, L_{total} - L_{n-1}) - I_r(L_n, L_{total} - L_n)\big)dr
\end{aligned}
$$

(26)

Let $L_{n-1} = a$, $L_{total} - L_n = b$.

Assume that, for a block of expected length k,

$$E(R) = \frac{L_{n-1} + L_n + 1}{2(L_{total} + 1)} = \frac{2a + k + 1}{2(a + b + k + 1)}$$

(25)

This is true for k = 1:

$$\int_0^1 r\big(I_r(a, b + 1) - I_r(a + 1, b)\big)dr = \int_0^1 r\left(I_r(a, b) + \frac{r^a(1 - r)^b}{bB(a, b)} - \left(I_r(a, b) - \frac{r^a(1 - r)^b}{aB(a, b)}\right)\right)dr$$
$$= \int_0^1 \frac{r^{a+1}(1 - r)^b}{B(a, b)}\left(\frac{1}{a} + \frac{1}{b}\right)dr = \frac{B(a + 2, b + 1)}{B(a, b)}\left(\frac{a + b}{ab}\right)$$
$$= \frac{B(a + 1, b + 1)}{B(a, b)}\frac{a + 1}{a + b + 2}\left(\frac{a + b}{ab}\right) = \frac{a + 1}{(a + b + 2)(a + b + 1)}$$

$$E(R) = \frac{a + b + 1}{1}.\frac{a + 1}{(a + b + 2)(a + b + 1)} = \frac{2a + 2}{2(a + b + 2)}$$

(27)

And also for $k = k + 1$:

$$E(R) = \frac{a + b + k + 1}{k + 1}\int_0^1 r\big(I_r(a, b + k + 1) - I_r(a + k + 1, b)\big)dr$$
$$= \frac{a + b + k + 1}{k + 1}\int_0^1 \left(r\big(I_r(a, b + k) - I_r(a + k, b)\big) + \frac{r^{a+1}(1 - r)^{b+k}}{(b + k)B(a, b + k)}\right.$$
$$\left. + \frac{r^{a+k+1}(1 - r)^b}{(a + k)B(a + k, b)}\right)dr$$
$$= \frac{a + b + k + 1}{k + 1}\frac{2a + k + 1}{2(a + b + k + 1)}.\frac{k}{a + b + k}$$
$$+ \frac{a + b + k + 1}{k + 1}\left(\frac{B(a + k + 2, b + 1)}{(a + k)B(a + k, b)} + \frac{B(a + 2, b + k + 1)}{(b + k)B(a, b + k)}\right)$$
$$= \frac{k}{k + 1}\left(\frac{2a + k + 1}{2(a + b + k)}\right)$$
$$+ \frac{a + b + k + 1}{k + 1}\left(\frac{B(a + k + 1, b + 1)}{(a + k)B(a + k, b)}\frac{a + k + 1}{a + b + k + 2}\right.$$
$$\left. + \frac{B(a + 1, b + k + 1)}{(b + k)B(a, b + k)}\frac{a + 1}{a + b + k + 2}\right)$$
$$= \frac{k(2a + k + 1)}{2(k + 1)(a + b + k)} + \frac{2b(a + k + 1) + 2a(a + 1)}{2(k + 1)(a + b + k)(a + b + k + 2)}$$
$$= \frac{k(2a + k + 1)(a + b + k + 2) + 2b(a + k + 1) + 2a(a + 1)}{2(k + 1)(a + b + k)(a + b + k + 2)}$$
$$= \frac{k(k + 1) + 2(k + 1) + 2a(k + 1)}{2(k + 1)(a + b + k + 2)} = \frac{2a + k + 2}{2(a + b + k + 2)}$$

(28)

Hence the expected location of the nth block of a Poisson-distributed multiblock copolymer is

$$E(R) = \frac{L_n + L_{n-1} + 1}{2(L_{total} + 1)}$$

(25)

for all integer values of L_n - L_{n-1}.

Variance and standard deviation of relative location. The variance of the relative location of the nth block is given by:

$$Var(R) = \frac{12L_{n-1}(L_{total} - L_n) + (l_n + 5)(l_n + 1)L_{total} - 2(l_n^2 - 1)}{12(L_{total} + 1)^2(L_{total} + 2)}$$

(29)

in which l_n is the expected length of the nth block, equal to $L_n - L_{n-1}$.

The standard deviation is the square root of the variance:

$$\sigma(R) = \frac{1}{L_{total} + 1}\sqrt{\frac{12L_{n-1}(L_{total} - L_n) + (l_n + 5)(l_n + 1)L_{total} - 2(l_n^2 - 1)}{12(L_{total} + 2)}}$$

(30)

When L_{total} is large with respect to l_n, this is approximately:

$$\sigma(R) \approx \frac{1}{L_{total}}\sqrt{\frac{H(L_{n-1}, L_n)}{2} + \frac{(l_n + 3)^2 - 4}{12}}$$

(31)

where $H(a,b)$ represents the harmonic mean of a and b (see Supplementary Table 1 for definition)

Proof. To obtain a formula for the standard deviation, we first evaluate $E(R^2)$.

$$E(R^2) = \frac{L_{total}}{L_n - L_{n-1}}\int_0^1 r^2\big(I_r(L_{n-1}, L_{total} - L_{n-1}) - I_r(L_n, L_{total} - L_n)\big)dr$$

Let $L_{n-1} = a$, $L_{total} - L_n = b$.

Assume that, for a block of expected length k,

$$E(R^2) = \frac{3a(a + k + 2) + (k + 1)(k + 2)}{3(a + b + k + 1)(a + b + k + 2)}$$

(32)

and hence

$$\int_0^1 r^2\big(I_r(a,b+k) - I_r(a+k,b)\big)dr = \frac{3a(a+k+2)+(k+1)(k+2)}{3(a+b+k+1)(a+b+k+2)}\frac{k}{a+b+k}$$

(33)

This is true for *k* = *1*:

$$\int_0^1 r^2\big(I_r(a,b+1) - I_r(a+1,b)\big)dr = \int_0^1 r^2\left(I_r(a,b)+\frac{r^a(1-r)^b}{bB(a,b)} - \left(I_r(a,b)-\frac{r^a(1-r)^b}{aB(a,b)}\right)\right)dr$$
$$= \int_0^1 \frac{r^{a+2}(1-r)^b}{B(a,b)}\left(\frac{1}{a}+\frac{1}{b}\right)dr = \frac{B(a+3,b+1)}{B(a,b)}\left(\frac{a+b}{ab}\right)$$
$$= \frac{B(a+2,b+1)}{B(a,b)}\frac{(a+2)}{a+b+3}\left(\frac{a+b}{ab}\right) = \frac{(a+2)(a+1)}{(a+b+3)(a+b+2)(a+b+1)}$$
$$= \frac{(a+2)!\,(a+b)!}{a!\,(a+b+3)!} = \frac{(a+1)(a+2)}{(a+b+1)(a+b+2)(a+b+3)}$$

$$E(R^2) = \frac{a+b+1}{1}\frac{(a+1)(a+2)}{(a+b+1)(a+b+2)(a+b+3)} = \frac{(a+1)(a+2)}{(a+b+1)(a+b+2)(a+b+3)}$$

(34)

And also for *k* = *k* + *1*:

$$E(R^2) = \frac{a+b+k+1}{k+1}\int_0^1 r^2\big(I_r(a,b+k+1) - I_r(a+k+1,b)\big)dr$$
$$= \frac{a+b+k+1}{k+1}\int_0^1 \left(r^2\big(I_r(a,b+k) - I_r(a+k,b)\big) + \frac{r^{a+k+2}(1-r)^b}{(a+k)B(a+k,b)} + \frac{r^{a+2}(1-r)^{b+k}}{(b+k)B(a,b+k)}\right)dr$$

(35)

Substituting $\int_0^1 r^2\big(I_r(a,b+k) - I_r(a+k,b)\big)dr$ gives

$$E(R^2) = \frac{k}{k+1}.\frac{3a(a+k+2)+(k+1)(k+2)}{3(a+b+k)(a+b+k+2)}$$
$$+\frac{a+b+k+1}{k+1}\left(\frac{B(a+k+3,b+1)}{(a+k)B(a+k,b)}+\frac{B(a+3,b+k+1)}{(b+k)B(a,b+k)}\right)$$
$$= \frac{k}{k+1}.\frac{(3a(a+k+2)+(k+1)(k+2))(a+b+k+3)}{3(a+b+k)(a+b+k+2)(a+b+k+3)}$$
$$+\frac{a+b+k+1}{k+1}\left(\frac{b(a+k+2)!\,(a+b+k-1)!}{(a+k)!\,(a+b+k+3)!}+\frac{(a+2)!\,(a+b+k-1)!}{(a-1)!\,(a+b+k+3)!}\right)$$

(36)

This can be rearranged to give

$$
\begin{aligned}
E(R^2) = &\frac{3ak(a+k+2)+k(k+1)(k+2)}{3(k+1)(a+b+k+2)(a+b+k+3)} \\
&+\frac{3ak(a+k+2)+k(k+1)(k+2)}{(k+1)(a+b+k)(a+b+k+2)(a+b+k+3)} \\
&+\frac{b(a+k+2)(a+k+1)+a(a+1)(a+2)}{(k+1)(a+b+k)(a+b+k+2)(a+b+k+3)} \\
&=\frac{3ak(a+1)}{3(k+1)(a+b+k+2)(a+b+k+3)}+\frac{3ak+k(k+2)}{3(a+b+k+2)(a+b+k+3)} \\
&+\frac{3ak(a+1)}{(k+1)(a+b+k)(a+b+k+2)(a+b+k+3)} \\
&+\frac{3ak+k(k+2)}{(a+b+k)(a+b+k+2)(a+b+k+3)} \\
&+\frac{ab(a+1)+a(a+1)(a+2)}{(k+1)(a+b+k)(a+b+k+2)(a+b+k+3)} \\
&+\frac{b(2a+k+2)}{(a+b+k)(a+b+k+2)(a+b+k+3)} \\
&=\frac{3ak(a+1)}{3(k+1)(a+b+k+2)(a+b+k+3)}+\frac{3ak+k(k+2)}{3(a+b+k+2)(a+b+k+3)} \\
&+\frac{a(a+1)(a+b+k+2(k+1))}{(k+1)(a+b+k)(a+b+k+2)(a+b+k+3)} \\
&+\frac{k(3a+b+k+2)+b(2a+2)}{(a+b+k)(a+b+k+2)(a+b+k+3)} \\
&=\frac{3ak(a+1)}{3(k+1)(a+b+k+2)(a+b+k+3)}+\frac{3ak+k(k+2)}{3(a+b+k+2)(a+b+k+3)} \\
&+\frac{3a(a+1)}{3(k+1)(a+b+k+2)(a+b+k+3)} \\
&+\frac{2(a+b+k)(a+1)}{(a+b+k)(a+b+k+2)(a+b+k+3)}+\frac{3k}{3(a+b+k+2)(a+b+k+3)} \\
&=\frac{3a(a+k+1)+6a+6+k^2+5k}{3(a+b+k+2)(a+b+k+3)}=\frac{3a(a+k+3)+(k+2)(k+3)}{3(a+b+k+2)(a+b+k+3)}
\end{aligned}
\tag{37}
$$

Hence,

$$
E(R^2)=\frac{3L_{n-1}(L_n+2)+(L_n-L_{n-1}+1)(L_n-L_{n-1}+2)}{3(L_{total}+1)(L_{total}+2)}
\tag{38}
$$

for positive integer values of L_n-L_{n-1}.

The variance can be calculated from:

$$Var(R) = E(R^2) - [E(R)]^2 = \frac{3a(a+k+2)+(k+1)(k+2)}{3(a+b+k+1)(a+b+k+2)} - \left(\frac{2a+k+1}{2(a+b+k+1)}\right)^2$$
$$= \frac{12a(a+k+2)+4(k+1)(k+2)}{12(a+b+k+1)(a+b+k+2)} - \frac{(12a^2+12a(k+1)+3(k+1)^2)(a+b+k+2)}{12(a+b+k+1)^2(a+b+k+2)}$$
$$= \frac{(12a^2+12a(k+1)+12a)+4(k+1)^2+4(k+1)-(12a^2+12a(k+1)+3(k+1)^2)}{12(a+b+k+1)(a+b+k+2)}$$
$$- \frac{(12a^2+12a(k+1)+3(k+1)^2)}{12(a+b+k+1)^2(a+b+k+2)}$$
$$= \frac{12a+(k+1)^2+4(k+1)}{12(a+b+k+1)(a+b+k+2)} - \frac{12a^2+12a(k+1)+3(k+1)^2}{12(a+b+k+1)^2(a+b+k+2)}$$
$$= \frac{12ab+(k+5)(k+1)(a+b)+(k+2)(k+1)^2}{12(a+b+k+1)^2(a+b+k+2)}$$

(39)

In terms of L_n, L_{n-1} and L_{total} this is:

$$Var(R) = \frac{L_{n-1}(L_{total}-L_n)}{(L_{total}+1)^2(L_{total}+2)} + \frac{(L_n-L_{n-1}+5)(L_n-L_{n-1}+1)(L_{total}-L_n+L_{n-1})}{12(L_{total}+1)^2(L_{total}+2)}$$
$$+ \frac{(L_n-L_{n-1}+2)(L_n-L_{n-1}+1)^2}{12(L_{total}+1)^2(L_{total}+2)}$$

(29)

Mode. The position with maximum probability (the mode) is given by

$$r_{max} = \frac{G(a, a+1, \dots, a+k-1)}{G(a, a+1, \dots, a+k-1) + G(b, b+1, \dots, b+k-1)}$$

(40)

where G(x_1, x_2, …, x_n) is the geometric mean of (x_1, x_2, …, x_n). Using the arithmetic mean A(x_1, x_2, …, x_n) as an approximation for the geometric mean yields

$$r_{max} \approx \frac{A(a, a+1, \dots, a+k-1)}{A(a, a+1, \dots, a+k-1) + A(b, b+1, \dots, b+k-1)} = \frac{2a+k-1}{2(a+b+k-1)} = \frac{L_{n-1}+L_n-1}{2(L_{total}-1)}$$

(41)

Proof. The mode is found by setting

$$\frac{\partial}{\partial r}[I_r(a, b+k) - I_r(a+k, b)] = \frac{r^{a-1}(1-r)^{b+k-1}}{B(a, b+k)} - \frac{r^{a+k-1}(1-r)^{b-1}}{B(a+k, b)} = 0$$

(42)

Since from the properties of the beta function

$$\frac{(1-r)^k}{B(a, b+k)} = \frac{r^k}{B(a+k, b)}$$

(43)

Substituting Supplementary Equation 43 in Supplementary Equation 42 gives:

$$r_{max} = \frac{B(a+k,b)^{\frac{1}{k}}}{B(a+k,b)^{\frac{1}{k}} + B(a,b+k)^{\frac{1}{k}}} = \frac{\sqrt[k]{\Gamma(b)\Gamma(a+k)}}{\sqrt[k]{\Gamma(b)\Gamma(a+k)} + \sqrt[k]{\Gamma(a)\Gamma(b+k)}}$$
$$= \frac{\sqrt[k]{a.(a+1)\dots(a+k-1)}}{\sqrt[k]{a.(a+1)\dots(a+k-1)} + \sqrt[k]{b.(b+1)\dots(b+k-1)}}$$
$$= \frac{G(a,a+1,\dots,a+k-1)}{G(a,a+1,\dots,a+k-1) + G(b,b+1,\dots,b+k-1)}$$

(40)

Median. The median value will fall between the expected value and the mode, and can thus be approximated by Supplementary Equation 44:

$$r_{median} \approx \frac{2a+k}{2(a+b+k)} = \frac{L_{n-1}+L_n}{2.L_{total}}$$

(44)

Maximum probability. For short blocks in a long polymer ($k << a, b$), Supplementary Equation 45 provides a reasonable approximation of the maximum probability (Supplementary Fig. 8).

$$P_{max}^{k} \approx \frac{k.\sqrt{a+b+k-1}}{\sqrt{2\pi(a+\frac{k-1}{2})(b+\frac{k-1}{2})}} = \frac{k}{\sqrt{\pi H(a+\frac{k-1}{2}, b+\frac{k-1}{2})}}$$

(45)

Derivation. The value of the maximum probability is

$$P_{max} = I_{r_{max}}(a,b+k) - I_{r_{max}}(a+k,b)$$

(46)

For $k = 1$, r_{max} is $a/(a + b)$, and the maximum probability is

$$P_{max}^{k=1} = I_{r_{max}}(a,b+1) - I_{r_{max}}(a+1,b) = \frac{{r_{max}}^{a}(1-r_{max})^{b}}{B(a,b)}\left(\frac{a+b}{ab}\right) = \frac{a^a b^b}{(a+b)^{a+b}}\frac{(a+b)!}{(a)!\,(b)!}$$

$$P_{max}^{k=1} \approx \frac{\sqrt{a+b}}{\sqrt{2\pi ab}} = \frac{1}{\sqrt{\pi H(a,b)}}$$

(47)

For short blocks in a long polymer ($k << a, b$), Supplementary Equation 45 provides a reasonable approximation of the maximum probability (Supplementary Fig. 8). This approximation is derived empirically from equation S56 and is presented without proof.

$$P_{max}^{k} \approx \frac{k.\sqrt{a+b+k-1}}{\sqrt{2\pi(a+\frac{k-1}{2})(b+\frac{k-1}{2})}} = \frac{k}{\sqrt{\pi H(a+\frac{k-1}{2}, b+\frac{k-1}{2})}}$$

(45)

Identities used in derivations.

Taylor series

$$e^{x} = \frac{1}{0\,!} + \frac{x}{1\,!} + \frac{x^{2}}{2\,!} + \cdots + \frac{x^{n}}{n!} + \cdots$$

(46)

$$xe^{x} = \frac{x}{0\,!} + \frac{x^{2}}{1\,!} + \frac{x^{3}}{2!} + \cdots + \frac{x^{n+1}}{n!} + \cdots$$

(47)

$$x^{2}e^{x} = \frac{x^{2}}{0\,!} + \frac{x^{3}}{1\,!} + \frac{x^{4}}{2!} + \cdots + \frac{x^{n+2}}{n!} + \cdots$$

(48)

$$\frac{d}{dx}(x^{2}e^{x}) = 2xe^{x} + x^{2}e^{x} = \frac{2x}{0\,!} + \frac{3x^{2}}{1\,!} + \frac{4x^{3}}{2!} + \cdots + \frac{(n+1)x^{n}}{(n-1)!} + \cdots$$

(49)

$$\frac{d^{2}}{dx^{2}}(x^{2}e^{x}) = 2e^{x} + 4xe^{x} + x^{2}e^{x} = \frac{2}{0\,!} + \frac{6x}{1\,!} + \frac{12x^{2}}{2!} + \cdots + \frac{n(n+1)x^{n-1}}{(n-1)!} + \cdots$$

(50)

$$2xe^{x} + 4x^{2}e^{x} + x^{3}e^{x} = \frac{2x}{0\,!} + \frac{6x^{2}}{1\,!} + \frac{12x^{3}}{2!} + \cdots + \frac{n(n+1)x^{n}}{(n-1)!} + \cdots$$

(51)

Stirling's approximation

$$x! \approx \sqrt{2\pi x}\left(\frac{x}{e}\right)^{x}$$

(52)

Identities involving beta functions

$$B(a+1,b) = \frac{aB(a,b)}{a+b} \tag{53}$$

$$B(a,b+1) = \frac{bB(a,b)}{a+b} \tag{54}$$

$$I_x(a+1,b) = I_x(a,b) - \frac{x^a(1-x)^b}{aB(a,b)} \tag{55}$$

$$I_x(a,b+1) = I_x(a,b) + \frac{x^a(1-x)^b}{bB(a,b)} \tag{56}$$

Supplementary Note 2. The Beta distribution as an approximation to ratio of two Poisson distributions.

No simple formula exists to calculate the ratio *X/(X+Y)* of two Poisson distributed variables, *X* and *Y*. If, however, *X* and *Y* are approximated by continuous variables ξ and η which follow a gamma distribution with the same mean and variance, their ratio, $\xi/(\xi + \eta)$, follows a beta distribution.

The gamma distribution is a two-parameter family of distributions defined on the domain [0,∞), characterized by a shape parameter, *k*, and a scale parameter, θ. Its probability distribution, mean and variance are given in Supplementary Equations 57-59.

$$\text{pdf: } f(x;k,\theta) = \frac{x^{k-1}e^{\frac{-k}{\theta}}}{\theta^k \Gamma(k)} \tag{57}$$

$$\text{mean: } E(X) = k\theta \tag{58}$$

$$\text{variance: } Var(X) = k\theta^2 \tag{59}$$

Thus a *Gamma(λ,1)* distributed variable has the same mean and variance as a *Poisson(λ)* distributed variable (Supplementary Fig. 9).

The ratio *X/X+Y* of two gamma-distributed variables with the same scale parameter, θ, and shape parameters α and β, respectively, has a *Beta(α,β)* distribution.

The probability density function and cumulative distribution function of a beta distribution are given by

$$\text{pdf: } f(x;\alpha,\beta) = \frac{x^{\alpha-1}(1-x)^{\beta-1}}{B(\alpha,\beta)}, 0 \leq x \leq 1 \tag{60}$$

$$\text{cdf: } F(x;\alpha,\beta) = \frac{\int_0^x t^{\alpha-1}(1-t)^{\beta-1}.dt}{B(\alpha,\beta)} = I_x(\alpha,\beta) \tag{61}$$

where $B(\alpha,\beta)$ and $I_x(\alpha,\beta)$ are the beta function and regularized incomplete beta function respectively (see Supplementary Table 1 for definitions).

If *X* and *Y* are approximated by the continuous gamma-distributed variables ξ and η, with equivalent means and variances, *X/(X+Y)* is approximated by $\xi/(\xi + \eta)$, which obeys a beta distribution (Supplementary Equations 62-65). Agreement between the discrete distribution *X/(X+Y)* and the continuous approximation, $\xi/(\xi + \eta)$, is very close, even for *E(X + Y)* as low as 10 (Supplementary Fig. 1).

$$X \sim Poisson(\lambda_X) \approx \xi \sim Gamma(\lambda_X, 1) \tag{62}$$

$$Y \sim Poisson(\lambda_Y) \approx \eta \sim Gamma(\lambda_Y, 1)$$

(63)

$$E(X) = Var(X) = E(\xi) = Var(\xi) = \lambda_X$$

(64)

$$\frac{X}{X+Y} \approx \frac{\xi}{\xi+\eta} \sim Beta(\lambda_X, \lambda_Y)$$

(65)

Supplementary Note 3. Analysis of chain distribution taking into account the possibility of looped chains.

The analysis of Fig. 4 neglected the possibility of forming looped structures, in which both maleimide units of the bismaleimide are found in the same chain. We feel justified in doing so as the bismaleimide contains a rigid bis(phenylene)methylene linker, which combined with the relatively rigid polystyrene chain, should disfavor loop formation. In addition, the experiment was carried out using short polystyrene chains and in the presence of a large excess of styrene relative to bismaleimide, so that steric hindrance and the absence of alternative substrates for reaction, which might otherwise favor backbiting, should not be an issue.

If the possibility of backbiting is taken into account, the peak in the chromatogram of Figure 4 which was ascribed to linear chains would also contain chains with one or more loops. Likewise, the double chains would include looped chains in addition to those containing a single maleimide linker.

If the probability that addition of a maleimide to a chain is followed by loop formation is ε, then the fraction of linear chains is equal to $P(0) + \varepsilon P(1) + \varepsilon^2 P(2) + \ldots$, where $P(k)$ is the probability that a chain adds k units of maleimide. The fraction of double chains (‘H’ in Figure 4) is given by $(1- \varepsilon)^2 P(1)^2 + 4\varepsilon^2(1- \varepsilon)^2 P(2)^2 + 4\,\varepsilon(1- \varepsilon)^2\,P(1).P(2) + \ldots$

The narrowest distribution consistent with an average of one maleimide per chain and a non-zero fraction, x, of chains without maleimide is given by $P(0) = x$, $P(1) = 1\text{-}2x$, $P(2) = x$, $P(\geq 3) = 0$. Assuming this distribution, the fraction of linear chains is $x + \varepsilon(1\text{-}2x)$, and the fraction of ‘H’ double chains is $(1\text{-}\varepsilon)^2(1\text{-}2x)^2 + 4\varepsilon^2(1- \varepsilon)^2 x^2 + 4\varepsilon(1- \varepsilon)^2\,x(1\text{-}2x)$. Solving these equations for the observed proportions of linear and ‘H’ chains, 34.4% and 15.1%, respectively, gives P(0) = 0.32, P(1) = 0.36, P(2) = 0.32, and ε = 0.06. The fraction of non-functional chains is still similar to that of the Poisson distribution (P(0) = 36.8%), and largely in excess of the 10% reported elsewhere.[S6,S7]

Supplementary Note 4. Effect of non-ideal dispersity on monomer distributions

The results presented in this paper are derived assuming ideal conditions of living polymerization: no side reactions such as chain transfer or termination; rapid initiation of all chains; and, in the case of RDRP, addition of a single monomer during each activation/deactivation cycle. Under these ideal conditions, chain lengths are Poisson distributed, with dispersity equal to $1 + 1/DP_n$.

In real polymerizations, higher dispersities are frequently observed, particularly for RDRP, where a variable number of monomers may be added during each activation/deactivation cycle. The following section briefly demonstrates the effect of increasing dispersity on the distribution of monomers in a multiblock copolymer (the styrene-maleimide copolymer of Figure 3 (top)).

For the purposes of the demonstration, it was assumed that the final multiblock copolymer had a dispersity of 1.10, at the lower end of typical values for RDRP (the reported dispersity in this case was 1.16). Three models were used to assign a dispersity to each block (Supplementary Table 3):

- Model 1: the dispersity of the polymer was assumed to remain constant at 1.10 throughout the polymerization – each segment is assigned a dispersity such that the polymer consisting of that segment plus all previous segments has a dispersity of 1.10.
- Model 2: the dispersity of each segment was assumed to be constant. Each segment was assigned a dispersity of 1.49 (1.50 for the final segment); the sum of all segments has a dispersity of 1.10.
- Model 3: the dispersity of each segment was given by the formula $Đ = 1 + a/DP_n$, where a is a constant (a = 5.25). The sum of all segments gives a polymer with dispersity 1.10.

In each case, if $Đ_A$ represents the dispersity of block A, and $Đ_B$ the dispersity of block B, the dispersity of the combined blocks A and B is calculated as $Đ_{A+B} = 1 + (\sigma_{A+B}/\mu_{A+B})^2 = 1 + (\sigma_A^2 + \sigma_B^2)/(\mu_A + \mu_B)^2 = 1 + [\mu_A^2(Đ_A - 1) + \mu_B^2(Đ_B - 1)]/(\mu_A + \mu_B)^2$.

The three models are not intended to be physically realistic but to cover a range of possible dispersity profiles. The evolution of the dispersity of the total polymer with each additional segment is shown in Supplementary Fig. 10.

For each model, a population of 1000 chains was simulated, assuming a negative binomial distribution for each segment. The parameters (p, r) of the distribution for each model are shown in Supplementary Table 3.

For each chain, the absolute and relative position of each interface between two successive segments was determined by summing the lengths of the preceding segments to give an absolute position, then dividing by the total chain length to obtain the relative position. Cumulative probability distributions were empirically generated for the position of each interface (the cumulative probability distributions for Model 1 are shown in Supplementary Fig. 11).

The empirically generated cumulative distribution functions were themselves modelled using negative binomial (absolute position) or beta distributions (relative position), with parameters determined by matching the moments of the distributions. Generally good agreement was obtained between the empirically generated and modelled distributions (Supplementary Fig. 11).

Finally, the distribution of each segment was obtained from the difference between successive cumulative probability distributions, following the same procedure as was used for the Poisson-distributed ideal polymer chains. The result for the case of Model 1 is shown in Supplementary Fig. 12.

The probability of finding a functional maleimide unit at any given position in the chain was then calculated for each polymer by multiplying the probability of finding a monomer from a given segment at any position in the chain by the probability that that monomer is a maleimide (assumed to be constant and equal to the average maleimide composition of the segment, e.g. 1/6 for segments 2 and 4, 1/3 for segment 6). The results for each of the three models are shown in Supplementary Fig. 13 (absolute distributions) and Supplementary Fig. 14 (relative distributions). The corresponding distributions, assuming Poisson-distributed segment lengths, are shown for comparison.

From Supplementary Figs. 13 and 14, it can be seen that, regardless of the details of the model used to allocate dispersities to each segment, a relatively small increase in the overall dispersity of the polymer from 1.02 (the Poisson limit) to 1.10 leads to substantial broadening of both absolute and relative position distributions of each segment.

Supplementary References

1 Gody, G., Maschmeyer, T., Zetterlund, P. B. & Perrier, S. Rapid and quantitative one-pot synthesis of sequence-controlled polymers by radical polymerization. *Nature Commun.* **4**, 3505 (2013).

2 Moatsou, D., Hansell, C. F. & O'Reilly, R. K. Precision polymers: a kinetic approach for functional poly(norbornenes). *Chem. Sci.* **5**, 2246-2250 (2014).

3 Fleury, G. & Bates, F. S. Structure and Properties of Hexa- and Undecablock Terpolymers with Hierarchical Molecular Architectures. *Macromolecules* **42**, 3598-3610 (2009).

4 Nagata, Y. *et al.* Preparation and Characterization of a Styrene−Isoprene Undecablock Copolymer and Its Hierarchical Microdomain Structure in Bulk. *Macromolecules* **38**, 10220-10225 (2005).

5 Zamfir, M. & Lutz, J.-F. Ultra-precise insertion of functional monomers in chain-growth polymerizations. *Nature Commun.*, **3**, 1138, (2012).

6 Lutz, J.-F., *et al.* Tailored Polymer Microstructures Prepared by Atom Transfer Radical Copolymerization of Styrene and N-substituted Maleimides. *Macromol. Rapid Commun.* **32**, 127-135 (2011).

7 Pfeifer, S. & Lutz, J.-F. Development of a Library of N-Substituted Maleimides for the Local Functionalization of Linear Polymer Chains. *Chem. Eur. J.* **14**, 10949-10957 (2008).
